# An Inflammatory Loop Between Spleen-Derived Myeloid Cells and CD4^+^ T Cells Leads to Accumulation of Long-Lived Plasma Cells That Exacerbates Lupus Autoimmunity

**DOI:** 10.3389/fimmu.2021.631472

**Published:** 2021-02-11

**Authors:** Eunkyeong Jang, Somi Cho, Sungjin Pyo, Jin-Wu Nam, Jeehee Youn

**Affiliations:** ^1^Laboratory of Autoimmunology, Department of Anatomy and Cell Biology, College of Medicine, Hanyang University, Seoul, South Korea; ^2^Department of Biomedical Science, Graduate School of Biomedical Science and Engineering, Hanyang University, Seoul, South Korea; ^3^Department of Life Science, College of Natural Sciences, Hanyang University, Seoul, South Korea

**Keywords:** long-lived plasma cell, myeloid-derived suppressor cell, autoantibody, lupus, sanroque mice

## Abstract

Splenic long-lived plasma cells are abnormally numerous and deleterious in systemic autoimmune diseases, yet how they accumulate remains poorly understood. We demonstrate here that a pathological role of spleen-derived CD11b^+^Gr-1^+^ myeloid cells (SDMCs) underpins the accumulation of splenic long-lived plasma cells in a lupus-prone model named sanroque. We found that SDMCs were progressively accumulated in sanroque mice from the early clinical phase. Transcriptome profiles revealed that SDMCs have a predominant shift toward an inflammatory phenotype relative to the bone marrow-derived counterparts and are distinct from neutrophils and monocytes. SDMCs were expanded *in situ* via splenic extramedullary myelopoiesis under the proinflammatory cytokine milieu during lupus progression. SDMCs promoted the development of IFN-γ-secreting Th1 and follicular helper T cells, thereby licensing CD4^+^ T cells to be pathologic activators of SDMCs and plasma cells. SDMCs also directly promoted the survival of plasma cells by providing B-cell activating factor of the TNF family. The frequency of SDMCs correlated with that of splenic long-lived plasma cells. Selective depletion of CD11b^+^Gr-1^+^ cells reduced autoantibody production in sanroque mice. Thus, our findings suggest that SDMCs expanded *in situ* establish a positive feedback loop with CD4^+^ T cells, leading to accumulation of long-lived plasma cells which exacerbates lupus autoimmunity.

## Introduction

Systemic lupus erythematosus (SLE) is a prototypic systemic autoimmune disease characterized by an abundance of Ig class-switched autoantibodies against nuclear components (1). Such autoantibodies target multiple organ systems, leading to chronic inflammation and, in severe cases, life threatening organ damage (2). In particular, high titers of IgG to double-stranded DNA (dsDNA) are associated with disease activity, severity, and lupus nephritis in more than half of SLE patients (3, 4). Plasma cells (PCs) that secrete high-affinity IgG autoantibodies are produced mainly during the germinal center (GC) response, which requires the activity of follicular helper T (Tfh) cells, in the spleen and other peripheral lymphoid tissues (5–7). Contradictory to conventional PCs (8, 9), we and others have shown that autoimmune long-lived PCs abnormally accumulate in the spleen rather than homing to the bone marrow (BM), as evidenced in murine models, such as the (NZBxNZW)F1, NZM2410, and K/BxNsf mice (10–12). This phenomenon is recapitulated in patients with autoimmune disease, such as primary warm autoimmune hemolytic anemia and primary immune thrombocytopenia (13, 14). Splenic long-lived PCs govern their own fate via a cell-autonomous mechanism and a positive feedback with Tfh cells, according to our previous studies (11, 15, 16). Importantly, they can transfer the disease to RAG knockout mice, indicating their pathogenic role (17), and are refractory to treatment with cytostatic drugs, leading to disease being incurable (9, 11, 12, 18). Therefore, the role of splenic long-lived PCs appears to be central to the exacerbation of autoantibody-mediated diseases, yet the mechanisms underlying their development and maintenance are poorly understood.

Another feature of systemic autoimmune diseases is the expansion of myeloid-derived suppressor cells (MDSCs), as shown by their abnormal accumulation in the spleens of murine models and in the peripheral blood of patients (19–23). MDSCs are a heterogeneous population of immature cells derived from myeloid progenitors, with potent immune regulatory activity (24, 25). They were first phenotypically identified in tumor-bearing mice by their expression of CD11b and Gr-1, but knowledge of their presence has been extended to other pathologic conditions including chronic inflammatory disorders. Murine MDSCs are subdivided into CD11b^+^Gr-1^hi^Ly6G^+^Ly6C^lo^ granulocytic or polymorphonuclear MDSCs (G- or PMN-MDSCs) and CD11b^+^Gr-1^lo^Ly6G^−^Ly6C^hi^ monocytic MDSCs (M-MDSCs). Although these phenotypes are also typical of neutrophils and monocytes, the functional features of MDSCs allow them to be distinguished from former (26, 27).

Many studies, mostly using tumor-bearing models, suggest that MDSCs accumulate via the same differentiation pathways in the BM as neutrophils and monocytes. Instead of differentiating into mature neutrophils and monocytes as they do during classical myelopoiesis, under the chronic inflammatory conditions of cancer, immature myeloid cells increase abnormally, are converted to MDSCs, and migrate to inflamed sites where they carry out their immunosuppressive activities (24). This abnormal process is known to be driven by two groups of interconnected signals (24, 28). The first group is important for expansion and mediated by myelopoietic factors, such as GM-CSF, G-CSF, M-CSF, and IL-6, and signals through STAT3, STAT5, IFN regulatory factor (IRF)-8, CCAAT/enhancer-binding protein (C/EBP)-β, and NOTCH. The second responsible for pathologic activation of MDSCs is triggered by proinflammatory cytokines, such as IFN-γ, IL-1β, IL-4, IL-6, IL-13, and TNF, and signals through NF-κB, STAT1, STAT3, and STAT6. However, it is unclear where the splenic MDSCs in autoimmune subjects originate and what factors contribute to their accumulation.

Although it was well-documented that MDSCs interfere with anti-tumor immunity by suppressing the proliferation of T cells, less is known about their role in autoimmune diseases. MDSCs have been found to attenuate disease severity in some studies with murine models and patients (19, 29–31), whereas others have reported a role of MDSCs in disease progression (21, 22, 32). At the cellular level, it is generally agreed that MDSCs target CD4^+^ T cells and suppress their proliferation and differentiation to Th1 and Tfh cells (20, 30, 32–34), although the effects of MDSCs on polarization toward Th17 cells is controversial (21, 22, 30, 32). Apart from the actions on T cells, MDSCs also interact with B cells to inhibit or, alternatively, promote the activation and differentiation of B cells into PCs (20, 31, 35–37). Despite many studies dealing with the multifaceted effects of MDSCs on T and B cells, there are no reports of crosstalk between MDSCs and PCs, which is plausible in the light of their close proximity in the autoimmune spleen. Therefore, the role of MDSCs in autoimmunity, especially as regards the development and survival of PCs, remains to be further clarified.

In this study, we investigated the role of splenic MDSCs in humoral autoimmunity using a lupus-prone strain named sanroque. The mouse is homozygous for the M199R “*san*” allele of *Roquin* gene (*Roquin*^*san*/*san*^), leading to spontaneous development of Tfh cells due to overexpression of the inducible T-cell costimulatory (ICOS) molecule (38). High affinity anti-nuclear IgG autoantibodies are detected in female sanroque mice as early as the preclinical phase, and their appearance is followed by the renal pathology typical of lupus (39). Here, we report that sanroque mice accumulate CD11b^+^Gr-1^+^ cells as well as long-lived PCs in their spleens during disease progression. The spleen-derived CD11b^+^Gr-1^+^ myeloid cells (referred to as SDMCs hereafter) are similar to MDSCs in terms of phenotype and suppression of T cell proliferation. Nevertheless, and surprisingly, SDMCs favor the differentiation of CD4^+^ T cells toward Th1 and Tfh cells, which promote the *in situ* activation of SDMCs and PCs, respectively. Moreover, SDMCs directly aid the survival of PCs by providing survival factors including B-cell activating factor of the TNF family (BAFF). Therefore, SDMCs and CD4^+^ T cells together establish an inflammatory positive feedback loop that contributes to the accumulation of splenic long-lived PCs with persistent autoantibody responses. Our results therefore confirm earlier studies reporting splenic myeloid cells intensify humoral autoimmunity, but extend that work by revealing novel roles of SDMCs in Th cells and PCs.

## Materials and Methods

### Mice

*Roquin*^*san*/+^mice on a C57BL/6 background were originally purchased from Mutant Mouse Regional Resource Center at the University of California at Davis. SKG mice on a BALB/c background were originally kindly donated by Dr. Shimon Sakaguchi (Osaka University, Japan). The mice were bred and maintained in a specific pathogen-free barrier facility at Hanyang University. C57BL/6 mice were purchased from Orient Bio (Seongnam-si, Korea). The *Roquin*^*san*/+^mice were intercrossed to generate homozygous *Roquin*^*san*/*san*^, namely sanroque mice. Female sanroque mice and their wild-type (WT) littermates were used at ~20 weeks of age unless indicated. To deplete CD11b^+^Gr-1^+^ cells, ~15 week-old sanroque mice were injected intraperitoneally with 10 mg/kg body weight gemcitabine (Sigma-Aldrich) or its vehicle PBS every 2 day over 7 days. Alternatively, SKG mice were injected intraperitoneally with anti-Gr-1 Ab (Bio X cell) or isotype-matched control Ab at a dose of 0.4 mg/mouse every day for 4 weeks starting at day 1 after curdlan administration (40). The protocol was approved by the Institutional Animal Care and Use Committee. All experiments in mice were conducted in strict accordance with guidelines and regulations.

### Histopathological and Immunohistochemical Examination

Mouse kidneys were fixed in 10% phosphate-buffered formalin, embedded in paraffin, sectioned at 5 μm thickness, and stained with H&E, as described (41). Glomerular size was measured using ImageJ software. Glomerular area was averaged from about 50 glomeruli from 3 independent sections. Spleens were cryo-sectioned and processed by standard fluorescence immunohistochemical methods, as described (41). Sections were stained with appropriate combinations of anti-B220-FITC, anti-B220-allophycocyanin, anti-CD4-FITC (all from eBioscience), anti-Gr-1-biotin (BD Biosciences), and streptavidin-Cy3 (Invitrogen). Fluorescence images were acquired using an LSM510 confocal microscope (Zeiss). To observe nuclear morphology, sorted SDMCs were smeared on slides by cytospin centrifugation, air-dried and stained with Diff-Quik solution (Sysmex Corporation). Images were acquired on an Olympus BX51 microscope equipped with an Olympus DP70 digital camera.

### FACS

Single cell suspensions of spleen and BM were prepared and assayed by FACS as described (42). To detect cytokine expression, CD4^+^ T cells were stimulated with 20 ng/ml PMA and 1 μM ionomycin (both from Sigma-Aldrich) in the presence of Golgi-stop reagent (BD Biosciences). For intracellular staining, cells were treated with Fixation/Permeabilization Solution (BD Biosciences). We used monoclonal antibodies (mAbs) to CD45 (30-F11), Ter119 (Ter119), CD11b (M1/70), Gr-1 (RB6-8C5), Ly6G (1A8), Ly6C (HK1.4), B220 (RA3-6B2), F4/80 (BM8), CD16/32 (2.4G2), CD34 (RAM34), Sca-1 (D7), CD117 (2B8), Flt3 (A2F10.1), CD4 (RM4-5. GK1.5), CD3 (145-2C11), CD8 (53-6.7), ICOS (C398.4A), BCL6 (K112-91), CD115 (T38-320), BAFF (121808), CD224 (A-4), MHC II (M5/114.15.2), CD80 (16-10A1), CD86 (GL1), CD138 (281-2), GL7 (GL7), FAS (Jo2), IFN-γ (XMG1.2), GM-CSF (MP1-22E9), IL-6 (MP5-20F3), IgG1 (A85-1; Santa Cruz), and MCL1 (REA924; Myltenyi Biotec), rat IgG2a (RTK2758), 7-aminoactinomycin D (7-AAD), and Annexin V (All from eBioscience or BD Biosciences unless indicated). Abs were conjugated with FITC, PE, PE-cy7, PerCP, allophycocyanin, or allophycocyanin-Cy7. Data were acquired with a FACS Canto II (BD Biosciences) and analyzed with Flowjo software (Tree Star Inc).

### Cell Isolation and Proliferation Assays

To isolate SDMCs, single cell suspensions of spleens from sanroque mice were stained with anti-CD11b and anti-Gr-1 mAbs and sorted by FACS Aria III (BD Biosciences). Purity routinely exceeded >97%. CD4^+^ T cells and CD19^+^ B cells from C57BL/6 mice were isolated by magnetic-activated cell sorting (MACS; Myltenyi Biotec) to >97% purity. After labeling with cell proliferation dye eFluor™ 670 (eBioscience), CD4^+^ T and CD19^+^ B cells were cocultured with SDMCs for 3 days in the presence of 5 μg/ml anti-CD3 plus 1 μg/ml anti-CD28 mAbs (eBioscience) and 10 μg/ml LPS (Sigma-Aldrich) plus 10 ng/ml IL-4 (BD Biosciences), respectively. In some experiments, transwells with 0.4 μm pores (Corning), 0.5 mM Nω-hydroxy-nor-arginine (nor-NOHA; Enzo Life Sciences), or 0.5 mM NG-monomethyl-l-arginine (L-NMMA; Enzo Life Sciences) were added to co-cultures. Cell proliferation levels are given as division indexes calculated with Flowjo software.

### CD4^+^ T Cell Differentiation

CD4^+^ T cells were stimulated with anti-CD3 and anti-CD28 mAbs in the presence or absence of SDMCs under Th1-polarizing conditions (10 ng/ml IL-12 and 10 μg/ml anti-IL-4 mAb) or Tfh-polarizing conditions (30 ng/ml IL-6, 50 ng/ml IL-21, 10 μg/ml anti-IL-12 mAb, 10 μg/ml anti-IFN-γ mAb, 10 μg/ml anti-IL-4 mAb, 5 μg/ml anti-IL-2 mAb, 20 μg/ml anti-TGF-β mAb). Reagents were from BD Biosciences, R&D Systems or Peprotech. To measure titers of IgM and IgG, Tfh-polarized CD4^+^ T cells were co-cultured with B cells sorted from C57BL/6 mice in the presence of 5 μg/ml anti-IgM (Jackson ImmunoResearch) and 10 ng/ml IL-4 for 6 days and the supernatants were assayed by ELISA.

### Evaluation of PC Apoptosis

CD3^−^CD11c^−^CD11b^−^B220^lo^CD138^+^ PCs from sanroque mice were sorted by FACS aria III to >98% purity and cultured with or without SDMCs at the indicated ratios in the presence of 1 μg/ml LPS. In some experiments, 10 μg/ml anti-BAFF mAb (AF2106; R&D Systems) or its isotype-matched control (AB-108-C; R&D Systems) was added. After 48 h, the cells were stained with 7-AAD and Annexin V and assayed by FACS, and the supernatants were collected to measure levels of total IgG by ELISA.

### Quantitative RT-PCR

Total RNA was purified from erythrocyte-depleted splenocytes or FACS-sorted SDMCs using TRIzol (Invitrogen). cDNA was generated using amfiRivert cDNA Synthesis Master Mix (GenDEPOT) and amplified by quantitative PCR using SYBR Green Quantitative PCR Master Mix (Applied Biosystems). mRNA levels were normalized to that of β-actin, and differences between samples and controls were calculated based on the 2^−ΔCT^ method. Primers for *Baff* were 5′-TGG AAT GGA TGA GTC TGC AA-3′ and 5′-ACA TCG CTG TGA AAC TGC TG-3″, and those for *Csf2, Ifng, Il6*, and *Actb* were as described (16, 43).

### RNA Sequencing (RNA-Seq) and Transcriptome Analysis

Total RNA was extracted from CD11^+^Gr-1^hi^ and CD11^+^Gr-1^lo^ cells from the spleens and BM of ~20 week-old sanroque mice. cDNA libraries were prepared using a TruSeq Stranded mRNA LT Sample Prep kit (Illumina) and sequenced on a NovaSeq 6000 platform using 101 bp paired-read strategy. RNA-seq data were obtained from two independent biological replicates per condition. RNA-seq data regarding classical and non-classical monocytes and neutrophils of C57BL/6 mice were downloaded from ArrayExpress (www.ebi.ac.uk/arrayexpress, accession number E-MTAB-8185). Raw sequencing reads were checked for quality using FastQC (version 0.11.8), filtered by Sickle (version 1.33) (44), aligned to the mouse genome GRCm38.p6 (GENCODE release M24) using STAR (version 2.5.3a) (45), and then quantified using featureCounts in the Subread package (version 1.6.4) (46). Transcripts per million (TPM) values of all genes in each sample were quantile-normalized to adjust variations among samples and used for downstream statistical analysis using edgeR (version 3.24.3) (47). Genes in which the sum of normalized TPM values are <10 were excluded from the analysis. *P*-values were adjusted using Benjamini and Hochberg's false discovery rate (FDR) correction. We considered genes with an adjusted *P* ≤ 0.05 and an at least 2-fold absolute expression difference between groups to be differentially expressed, unless otherwise indicated. For principal component assay (PCA) the top 1,000 variable genes were analyzed using R in the FactoMineR package (48). Gene set enrichment analysis (GSEA) used on 50 different hallmark gene set terms from Molecular Signatures Database (MSigDB v7.1) was conducted with GSEA v4.0.3 (49, 50). Enrichment scores were determined using Weighted Kolmogorov-Smirnov Statistic.

### ELISA and ELISPOT Assays

Concentrations of total IgG and total IgM Abs were measured using ELISA kits (Bethyl Laboratories and Alpha Diagnostic International, respectively), according to the manufacturer's protocols. The titers of serum anti-dsDNA IgG Ab and numbers of anti-dsDNA IgG-secreting cells in spleens and BM were determined as described previously (42). To measure levels of BAFF in sera and in supernatants of SDMCs cultured with 2 μg/ml LPS for 48 h, samples were assayed using Duoset ELISA kit (R&D Systems).

### Statistics

Statistical comparisons were made by unpaired or paired Student′s *t*-tests using Graph Pad Prism 7 software (Graph Pad Software Inc.). Correlation analysis was analyzed by the Spearman rank correlation test. Two-tailed *P* < 0.05 were considered significant.

## Results

### Accumulation of Myeloid Lineage Cells in the Spleens of Sanroque Mice

**Figure 1:**
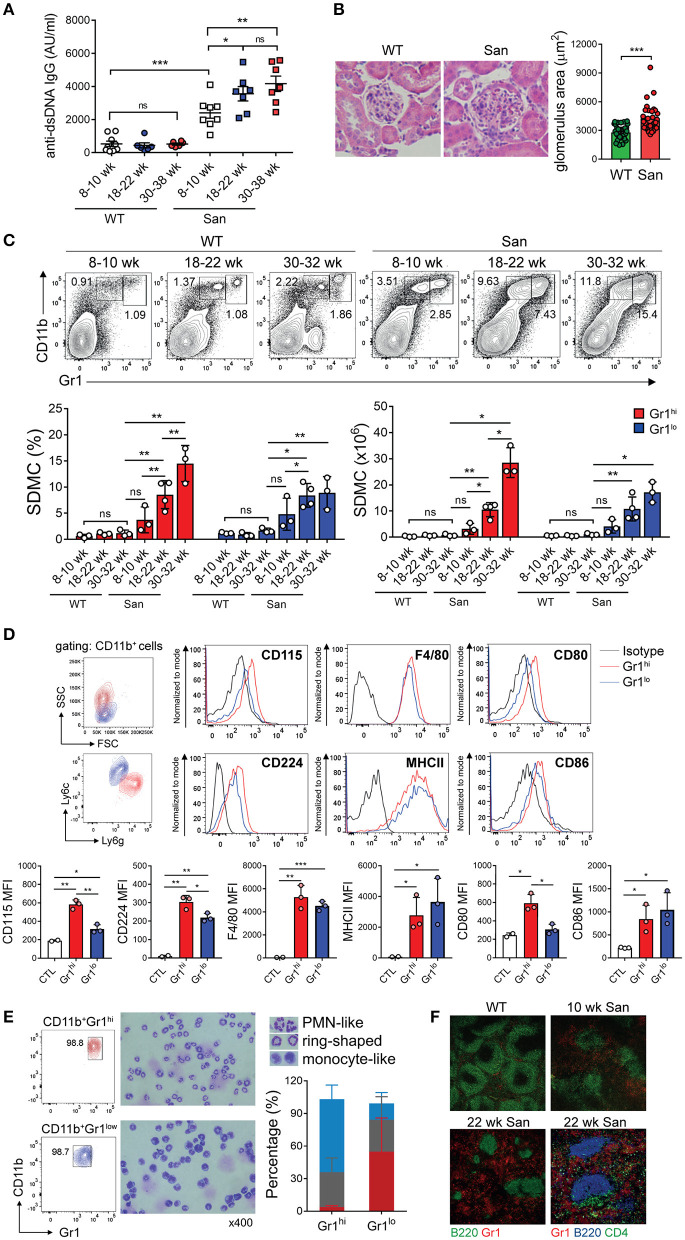
Accumulation of myeloid cells in sanroque mice. **(A)** Sera from sanroque mice and WT littermates at the indicated ages were assayed by ELISA to measure anti-dsDNA IgG titers. **(B)** Kidney sections of 18–22 week-old mice were stained with H&E and assayed by histopathological methods. **(C,D)** Spleen cells from sanroque and WT mice were assayed by FACS. FACS profiles were gated on whole live cells **(C)** and CD11b^+^ cells **(D)**, and percentages of cells within the indicated areas are shown. **(E)** CD11b^+^Gr-1^hi^ and CD11b^+^Gr-1^lo^ cells sorted from the spleens of sanroque mice were stained and enumerated according to nuclear morphology. PMN-like, blue; ring-shaped, gray; monocyte-like, red. **(F)** Spleens from ~10 and 22 week-old sanroque and 10 week-old WT mice were examined by fluorescence confocal microscopy. Original magnification, ×100 or ×200 (the lower right image). All graphs show means ± SDs, with symbols representing the values of individual mice. The data are pooled from 3 independent experiments **(A)** or are representative of at least 3 independent experiments **(B–F)**. *p < 0.05, **p < 0.01, and ***p < 0.001 by Student's *t*-test. ns, not significant; San, sanroque; AU, arbitrary unit.

Sanroque mice have been shown to spontaneously produce anti-dsDNA IgG Ab, which correlates with disease activity and lupus nephritis. The time of disease onset and kinetics of disease progression vary depending on the experimental settings (51). In our system, female sanroque mice had significantly elevated titers of anti-dsDNA IgG Ab compared to their WT littermates, without displaying any overt pathologic symptoms at 8–10 weeks of age (Figure 1A). The titer of anti-dsDNA IgG Ab was significantly elevated in sanroque, but not WT, mice at 18–22 weeks of age, at which time signs of lupus nephritis, such as enlarged glomeruli, hypercellularity and lymphocyte infiltration, were evident (Figure 1B and not shown). The high Ab titer and histopathological manifestations were sustained up to > 30 weeks of age. Therefore, age 18–22 weeks seemed to correspond to the early clinical phase for female sanroque mice in our system.

Given that other lupus-prone mice, such as MRL^*lpr*^ and (NZBxNZW)F1, have abnormal levels of CD11b^+^Gr-1^+^ myeloid cells in their spleens during disease progression, we examined whether this was also the case in sanroque mice. We observed progressive expansion of spleen-derived CD11b^+^Gr-1^+^ myeloid cells (i.e., SDMCs) in sanroque mice but not in WT littermates (Figure 1C). These cells were sorted by FACS into two subsets, CD11b^+^Gr-1^hi^ and CD11b^+^Gr-1^lo^ cells. The difference in the frequencies of both subsets between sanroque and WT mice increased with age, and reached statistical significance from 18 to 22 week, i.e., in the early clinical phase. The phenotype of the CD11b^+^Gr-1^hi^ cells with high side scatter was Ly6G^hi^Ly6C^lo^CD115^hi^CD224^hi^F4/80^+^MHCII^+^CD80^+^CD86^+^, which is similar to that of G-MDSCs and distinct from that of neutrophils (CD115^−^CD224^−^F4/80^−^MHCII^−^) (Figure 1D). The phenotype of the CD11b^+^Gr-1^lo^ cells with low side scatter was Ly6G^lo^Ly6C^hi^CD115^lo^CD224^lo^ F4/80^+^MHCII^+^CD80^−^CD86^+^, which is reminiscent of M-MDSCs. The CD11b^+^Gr-1^hi^ cell population was composed of ~64% cells with polymorphic segmented nuclei, ~31% cells with ring-shaped nuclei, and 5% cells with monocyte-like nuclei (Figure 1E). In contrast, the majority of the CD11b^+^Gr-1^lo^ cell fraction had a monocyte-like appearance, with a minority of cells with polymorphic segmented or ring-shaped nuclei. Consistently, Gr-1^+^ cells were more numerous in the spleens of sanroque mice than in those of the WT controls (Figure 1F). These cells were widely detected in extrafollicular region of sanroque spleens without selective perifollicular topography shown in WT spleens. Overall, in terms of composition, phenotype, and localization, the CD11b^+^Gr-1^hi^ and CD11b^+^Gr-1^lo^ cell fractions seemed to fit the criteria of G-MDSC and M-MDSC, respectively. Therefore, these results indicate that autoimmunity-driven pathology is associated with expansion of G-MDSC- and M-MDSC-like cells in the spleens of sanroque mice, and suggest that these SDMC populations are implicated in the persistence of autoimmunity in sanroque mice.

### Transcriptome Profiles Identify SDMCs as MDSCs With a Predominant Shift Toward an Inflammatory Phenotype

To obtain a more comprehensive understanding of the SDMCs that expand during autoimmune disease progression, we conducted an RNA-seq analysis of CD11b^+^Gr-1^hi^ and CD11b^+^Gr-1^lo^ cells sorted from the spleens of female sanroque mice aged ~20 weeks, i.e., in the early clinical phase. Autologous BM cells with the same phenotypes, which correspond to prototypic G- and M-MDSCs, were used as reference.

**Figure 2:**
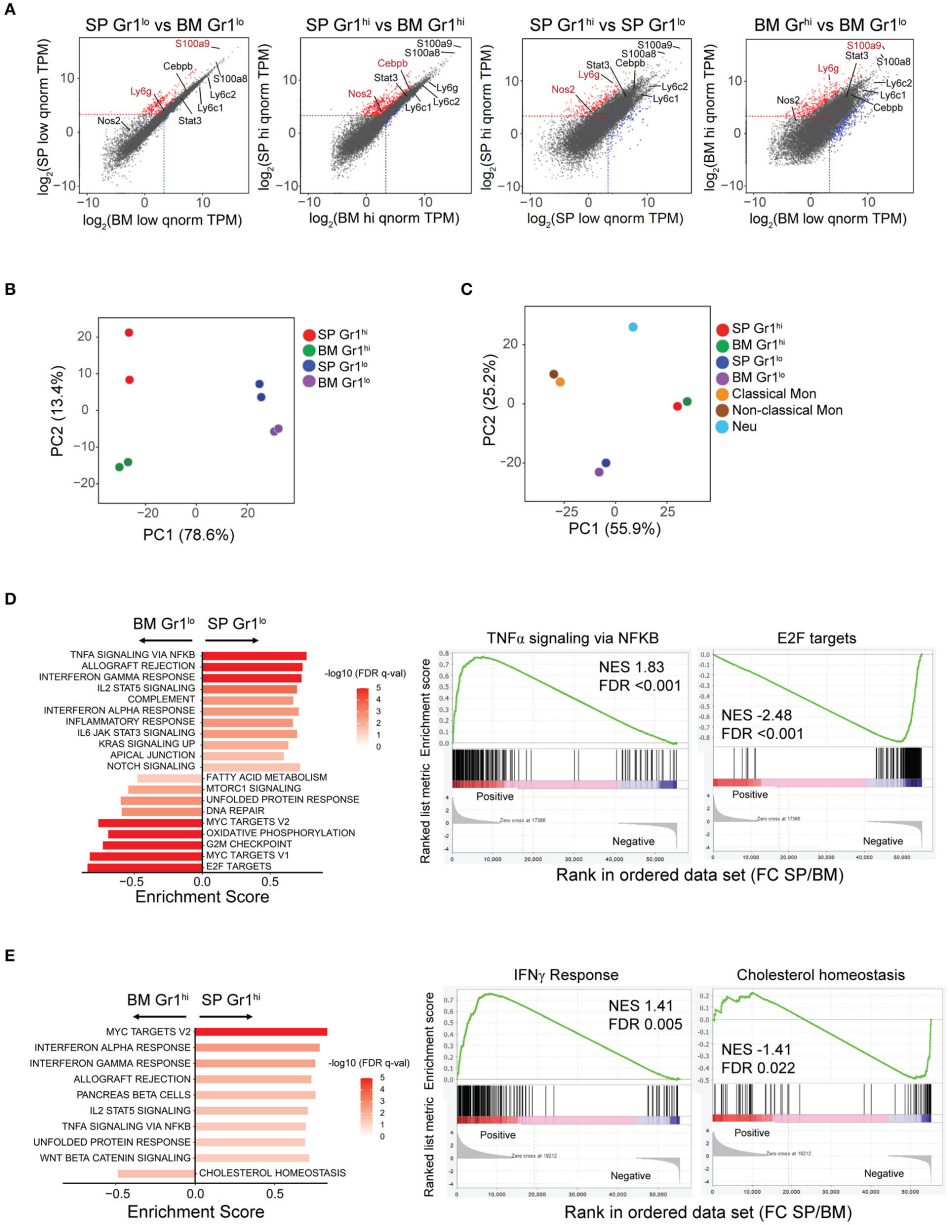
Transcriptome profiling of SDMCs. RNA-seq analysis of CD11b^+^Gr-1^hi^ and CD11b^+^Gr-1^lo^ cells from spleen and BM of sanroque mice. **(A)** The transcriptomes of the 4 sorted cell populations are displayed as pairwise scatter plots. Upregulated and downregulated genes under the criteria (Log_2_FC > 1 and adjusted *P* < 0.05 for the left two plots; Log_2_FC > 3 and adjusted *P* < 0.01 for the right two plots) are displayed as red- and blue-colored dots, respectively. Signature genes of MDSCs are indicated. **(B,C)** PCA of gene expression in the 4 cell populations without **(B)** and with other sources of neutrophils and monocytes **(C)**. Symbols represent individual samples, colored by population. **(D,E)** GSEA of gene expression comparing spleen- vs. BM-derived CD11b^+^Gr-1^lo^ subsets **(D)** and spleen- vs. BM-derived CD11b^+^Gr-1^hi^ subsets **(E)**. Data of significantly enriched gene set terms (left panels) and representative enrichment plots (right panels) are shown. FDR q-values are indicated as red-white colors. NES, normalized enrichment score; FDR, false discovery rate.

Transcriptome profiles differed across all four subsets (Figure 2A). We identified 277 differentially expressed genes (DEGs) comprising 262 upregulated and 15 downregulated genes, with a fold change (FC) >2 between spleen- and BM-derived CD11b^+^Gr-1^lo^ subsets. DEGs between spleen- and BM-derived CD11b^+^Gr-1^hi^ subsets was 2.2-fold more numerous, with 535 upregulated and 53 downregulated genes under the same criteria. Despite hundreds of DEGs, many MDSC signature genes, such as *Ly6c, Ly6g, Stat3*, and *S100a8*, were not included in the DEGs, although *S100a9, Nos2*, and/or *Cebpb* were upregulated. As expected, DEGs were most numerous in BM-derived CD11b^+^Gr-1^lo^ cells compared to BM-derived CD11b^+^Gr-1^hi^ cells (411 upregulated and 358 downregulated, with FC > 8 and adjusted *P* < 0.01). However, interestingly, DEGs between the two spleen-derived subsets were ~2-fold-fewer than those between two BM-derived subsets, implying functional convergence of the two subsets of SDMCs.

PCA separated all four subsets (Figure 2B). According to PC1, the spleen-derived CD11b^+^Gr-1^hi^ and CD11b^+^Gr-1^lo^ cells seemed most similar to BM-derived CD11b^+^Gr-1^hi^ and CD11b^+^Gr-1^lo^ cells, respectively. The distance between the two spleen-derived subsets was shorter than that between the two BM-derived subsets, supporting the idea of functional convergence. When compared to the transcriptomes of neutrophils and classical or non-classical monocytes with identical genetic backgrounds to the sanroque mice, all four subsets were segregated from neutrophils and monocytes (Figure 2C).

To assess the biological processes and signaling pathways significantly enriched in SDMCs relative to their BM-derived counterparts, we performed a GSEA. Gene sets related to proinflammatory signals, such as the pathways mediated by IFN-γ, IFN-α, TNF-α-NF-κB, IL-6-JAK-STAT3, IL-2-STAT5, and NOTCH, were highly enriched in spleen-derived CD11b^+^Gr-1^lo^ cells compared to BM-derived CD11b^+^Gr-1^lo^ cells (Figure 2D, Supplementary Figure 1). Similarly, the gene sets enriched in spleen-derived CD11b^+^Gr-1^hi^ cells compared to their BM-derived counterparts included genes related to signaling pathways mediated by IFN-γ, IFN-α, TNF-α-NF-κB, IL-2-STAT5, NOTCH, and WNT, and unfolded protein responses (Figure 2E, Supplementary Figure 1). Interestingly, gene sets related to proliferation (E2F targets, Myc targets, and DNA repair) and metabolic reprogramming (oxidative phosphorylation, fatty acid metabolism, cholesterol homeostasis, and mTORC1 signaling) were less activated in spleen-derived CD11b^+^Gr-1^lo^ or Gr1^hi^ cells than in their BM-derived counterparts.

We further assessed whether proinflammatory molecules are indeed upregulated in SDMCs. Consistent with RNA-seq data, subsets of SDMCs expressed higher levels of IL-18 than their BM counterparts, and this phenomenon was more prominent in the Gr-1^lo^ subset (Supplementary Figure 2A). The expression of IL-β, TNF, IFN-α and IL-6 was significantly increased in SDMCs, compared to autologous B cells, with different dominance between subsets. *In vivo* provision of IL-1β and IFN-α was dominated by the Gr-1^hi^ subset, while IL-6 expression was dominated by the Gr-1^lo^ subset (Supplementary Figures 2B–D). TNF was upregulated evenly in both subsets (Supplementary Figure 2B). Interestingly, IFN-α expression was highly induced in Gr-1^lo^ SDMCs upon PMA stimulation. Among costimulators important for the priming of CD4^+^ T cells, ICOSL, but not CD40, was upregulated on both subsets of SDMCs (Supplementary Figure 2E and not shown).

These results taken together identify spleen-derived CD11b^+^Gr-1^lo^ and CD11b^+^Gr-1^hi^ cells as *bona fide* G- and M-MDSCs, respectively, that are distinct from neutrophils and monocytes. Nevertheless, SDMCs seem to evolve differently from BM-derived MDSCs, with a predominant shift toward more proinflammatory and less proliferative phenotype. This process may lead two subsets of SDMCs to functionally converge.

### *In situ* Generation and Proinflammatory Activation of SDMCs in Sanroque Mice

We wanted to know where SDMCs originated. According to previous reports, MDSCs are expanded in the BM via abnormal myelopoiesis. Long term-hematopoietic stem cells (LT-HSCs) in the BM develop first into short term (ST)-HSC and then via multipotent progenitor (MPP) stages into lineage-restricted progenitors, i.e., lymphoid progenitors, granulocyte-macrophage progenitors (GMPs), and megakaryocyte-erythrocyte progenitors (MEPs) (52). GMPs further differentiate through several stages into MDSCs under certain pathologic conditions (24).

To examine the possibility that SDMCs emerge in the BM and then migrate to the spleen, as suggested previously, we examined the composition of cells harbored in the BM of disease-established sanroque mice. Unexpectedly, the frequencies of MPPs and GMPs did not differ significantly in sanroque mice, although the frequency of earlier progenitor LT-HSCs was elevated (Supplementary Figure 3). Numbers of total BM cells and CD11b^+^Gr-1^hi^ and CD11b^+^Gr-1^lo^ cells did not differ between sanroque and WT mice. Therefore, it was not evident whether myelopoiesis was enhanced in the BM of sanroque mice.

**Figure 3:**
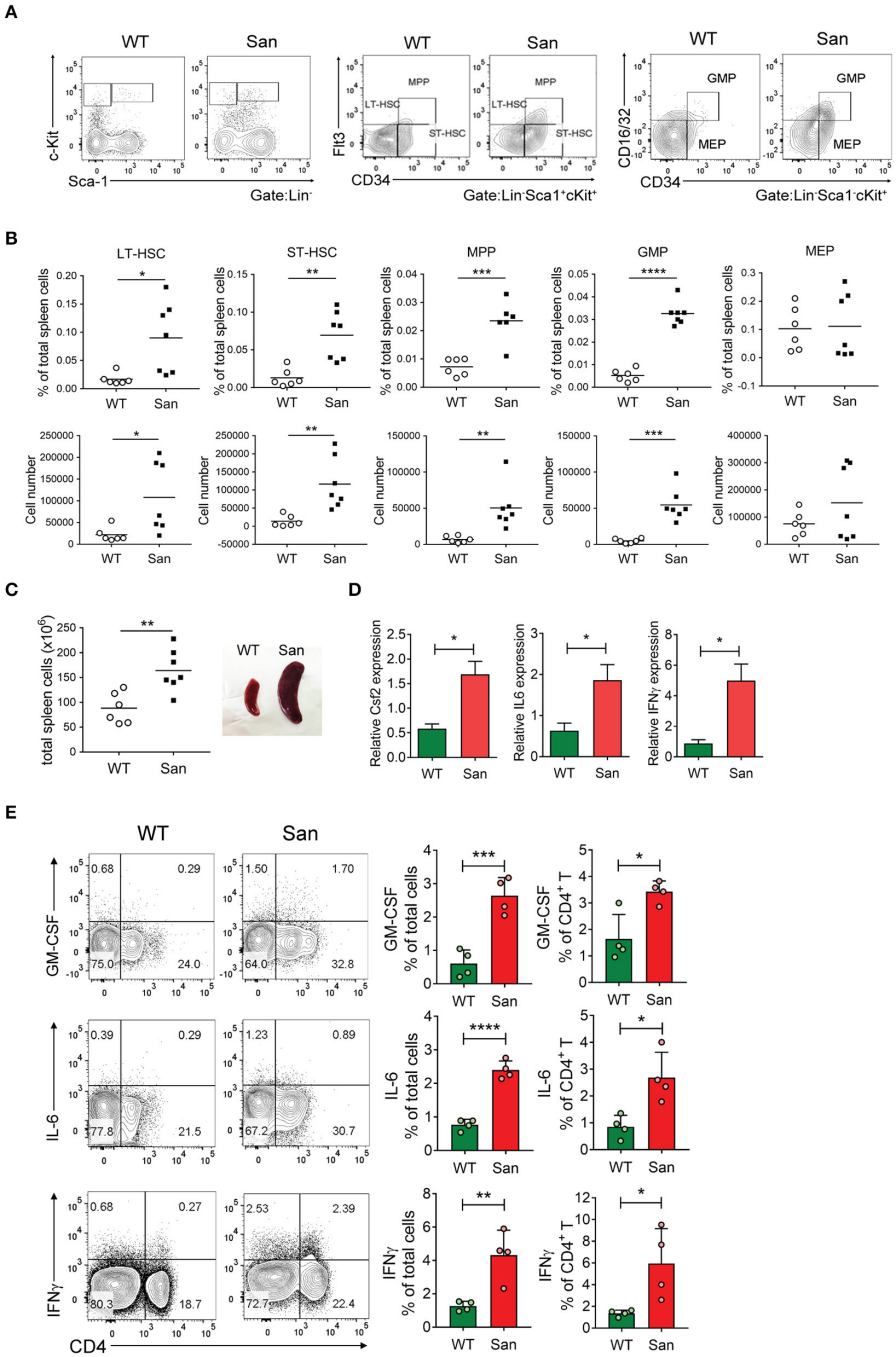
Splenic extramedullary development of SDMCs in sanroque mice. **(A,B)** Spleen cells from sanroque mice and their WT littermates were assayed by FACS. Representative FACS profiles **(A)** and percentages and numbers of the indicated cell populations with symbols representing the values of individual mice and horizontal bars representing means **(B)** are shown. **(C)** Numbers of total spleen cells and a representative photograph displaying spleens from WT and sanroque mice are shown. **(D)** Spleen cells were assayed by quantitative RT-PCR. **(E)** Spleen cells were stimulated with PMA and ionomycin for 5 h and assayed by intracellular FACS. Representative FACS profiles gated on whole live cells are shown along with data displayed as means ± SDs with symbols representing the values of individual mice. Data are representative of 2 independent experiments. *p < 0.05, **p < 0.01, ***p < 0.001, and ****p < 0.0001 by Student's *t*-test. San, sanroque.

This result led us to examine the possibility of splenic extramedullary expansion of SDMCs. Surprisingly we found that the proportions and absolute numbers of GMPs and their upstream progenitors from LT-HSC to MPPs were significantly higher in sanroque mice than in WT controls. In contrast, the proportions and absolute numbers of MEPs did not differ (Figures 3A,B), indicating that the developmental pathway leading to GMPs is favored over that leading to MEPs at the lineage bifurcation point in sanroque mice. The expression level of CD115 did not significantly differ in GMPs from WT and sanroque mice (Supplementary Figure 3D). Total spleen cells were ~2-fold more numerous, and this was mirrored by splenomegaly (Figure 3C). These findings reveal that splenic extramedullary myelopoiesis is highly activated in the spleen of sanroque mice and suggest that the accumulation of SDMCs takes place *in situ*.

Next, we asked which factors contributed to the accumulation of SDMCs in the spleens of sanroque mice. According to our transcriptome analysis, many genes that encode factors previously identified to expand and activate BM-derived MDSCs were preferentially activated in SDMCs. For example, expression of genes encoding TNF, IL-1β, M-CSF (*Csf1*), receptors for G-CSF (*Csf3r*), M-CSF (*Csf1r*), IFN-γ, TNF, IL-4 and IL-13, NF-κB, IRF8, and C/EBPβ was significantly upregulated in CD11b^+^Gr-1^lo^ and/or Gr-1^hi^ SDMCs (Supplementary Figures 1, 2). This supported the hypothesis regarding the *in situ* generation of SDMCs. This also suggested that extrinsic factors produced by cells other than SDMCs participate in the process.

We sought to identify such extrinsic factors enriched in the spleen of sanroque mice, and found that transcript levels of GM-CSF, IL-6, and IFN-γ were significantly higher in the spleens of sanroque mice than in those of WT mice (Figure 3D). The frequencies of cells producing these cytokines were also higher, and CD4^+^ T cells were major sources of these cytokines (Figure 3E). These results suggest that the autoimmune spleen contains cells (especially CD4^+^ T cells) that secrete factors required for the expansion and/or activation of SDMCs and thereby establishes a niche for these cells.

### SDMCs From Sanroque Mice Promote the Differentiation of CD4^+^ T Cells Toward Th1 and Tfh Cells

**Figure 4:**
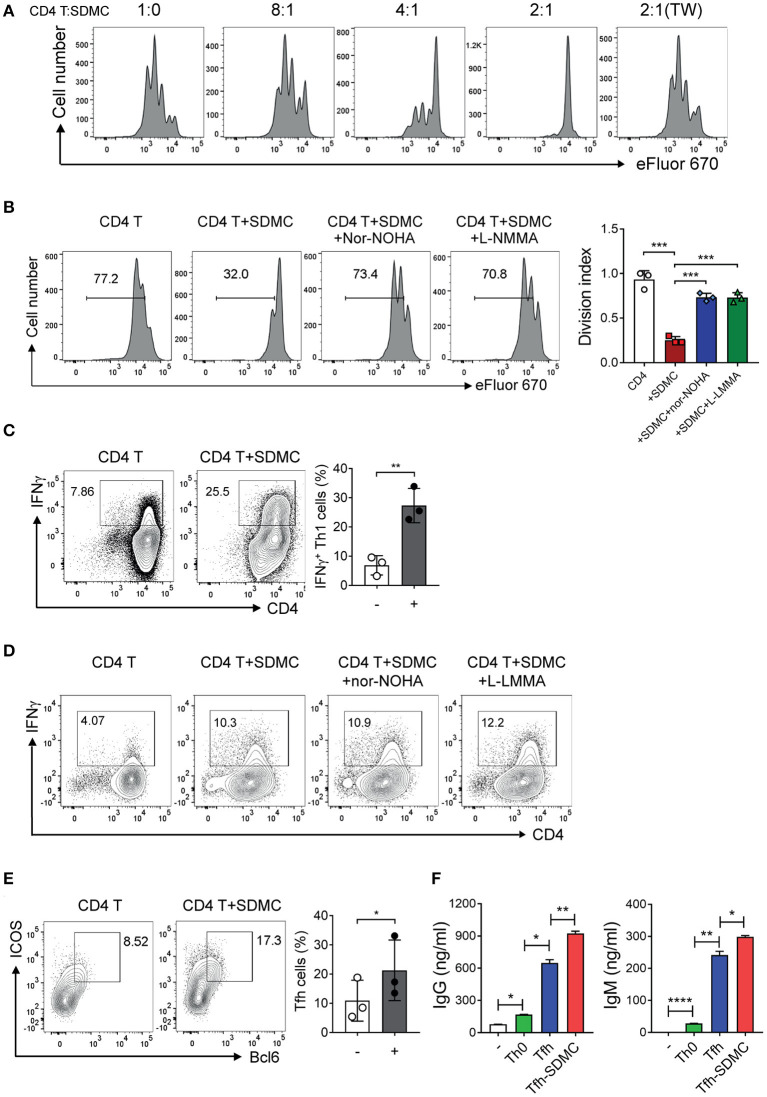
Effects of SDMC on the proliferation and differentiation of CD4^+^ T cells. **(A,B)** CD4^+^ T cells stained with eFluor 670 were stimulated with anti-CD3 and anti-CD28 mAbs in the presence or absence of SDMCs from sanroque mice at the indicated ratios **(A)** and a 2:1 ratio of CD4^+^ T cells: SDMCs **(B)**. In some wells, transwells (TW) were used to separate SDMCs and CD4^+^ T cells **(A)** and inhibitors were added as indicated **(B)**. **(C–E)** CD4^+^ T cells were cultured under Th1- **(C,D)** or Tfh-polarizing conditions **(E)** in the presence or absence of SDMCs and inhibitors, and assayed by intracellular FACS. **(F)** CD4^+^ T cells treated as in E were co-cultured with syngeneic B cells in the presence of anti-IgM and IL-4 and culture supernatants were assayed by ELISA. Data are displayed as the means ± SDs with symbols representing the values of individual mice and are representative of 2 independent experiments. *p < 0.05, **p < 0.01, ***p < 0.001, and ****p < 0.0001 by Student's *t*-test.

The canonical feature of MDSCs is suppression of T cell proliferation through inducible nitric oxide synthase (iNOS) and/or arginase 1-dependent pathways. This applies also to most cases including even MDSCs, which have additional functions other than immune suppression (20, 21, 32). To test whether SDMCs from sanroque mice fit this functional definition, we sorted CD11b^+^Gr-1^+^ cells from the spleens of ~20-week-old sanroque mice, co-cultured them with syngeneic CD4^+^ T cells under TCR/CD28 stimulation, and measured their proliferation. The presence of CD11b^+^Gr-1^+^ cells was found to inhibit the proliferation of the CD4^+^ T cells in a cell number- and contact-dependent manner (Figure 4A). This suppressive effect was almost completely abrogated by treatment with either iNOS inhibitor or arginase-1 inhibitor (Figure 4B). When splenic dendritic cells were added to the culture instead of SDMCs, the proliferation of CD4^+^ T cells was not affected (Supplementary Figure 4). Therefore, the SDMCs from disease-established sanroque mice seem to be *bona fide* MDSCs.

Next, we tested the effect of SDMCs on the differentiation of CD4^+^ T cells into Th effector subsets. The presence of SDMCs favored the development of CD4^+^ T cells toward IFN-γ-producing Th1 cells under Th1-polarizing conditions (Figure 4C). This effect was not altered by the addition of inhibitors of iNOS and arginase-1, indicating that the process is independent of iNOS and arginase-1 (Figure 4D). SDMCs slightly increased the fraction of Th1 cells even under the non-polarizing conditions (only containing anti-CD3 and anti-CD28 Abs) and this effect was abolished by addition of neutralizing Ab to IL-18, indicating the involvement of IL-18 in SDMC-mediated Th1 induction (Supplementary Figure 5). SDMCs had little or no effect on the differentiation of Th2 and Th17 cells (data not shown). Importantly, CD4^+^ T cells cultured in the presence of SDMCs gave rise to more numerous ICOS^+^BCL6^+^ Tfh cells than those cultured in the absence of SDMCs (Figure 4E). Consistent with this, co-culture of CD4^+^ T cells with SDMCs led them to more efficiently help B cells to differentiate into CD138^+^ PCs, and to secrete higher levels of IgM and IgG (Supplementary Figure 6, Figure 4F). These results demonstrate that SDMCs promote the development of CD4^+^ T cells to functional Tfh cells.

To determine whether the above effect of SDMCs is recapitulated in other autoimmune models, we carried out experiments using MRL^lpr^ and SKG strains. The former has been shown to accumulate SDMCs, but their roles in autoimmunity remain to be clarified. We found that SDMCs sorted from MRL^lpr^ mice during lupus progression were able to suppress the proliferation of autologous CD4^+^ T cells and to promote their polarization toward Th1 cells (Supplementary Figure 7). The SKG strain is an autoimmune arthritis model, in which Tfh cells as well as Th17 cells are pathogenic (53, 54), yet SDMCs remain to be identified. We found that SDMCs were highly accumulated in the spleen of arthritis-established SKG mice and could promote the polarization of CD4^+^ T cells toward Th1 and Tfh cells (Supplementary Figure 9). Thus, the effects of SDMCs on promoting Th1 and Tfh cells seem to be a common feature of SDMCs in autoimmune mice.

### SDMCs Promote B Cell Activation and PC Survival

**Figure 5:**
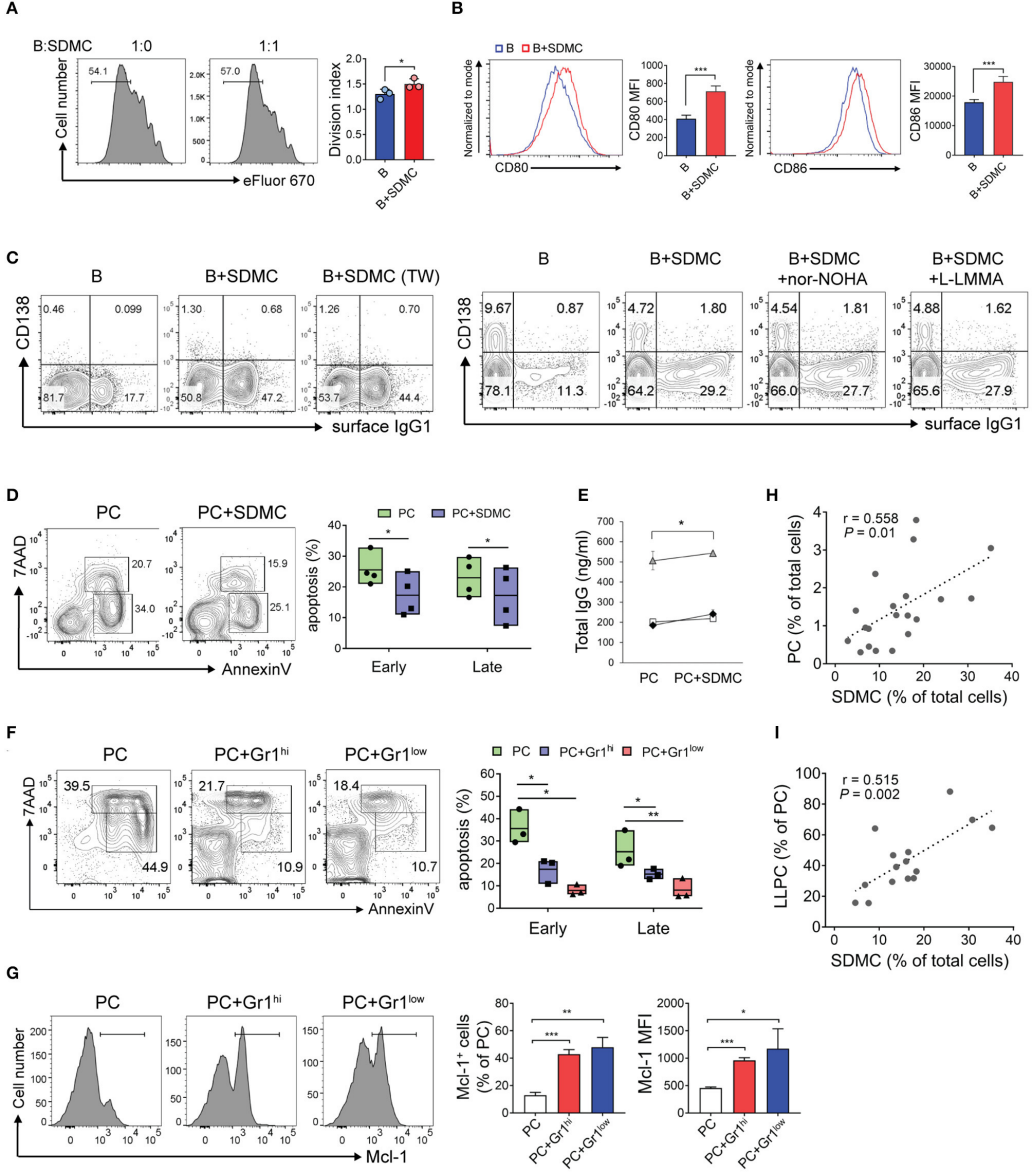
Effects of SDMCs on activation of B cells and survival of PCs. **(A–C)** CD19^+^ B cells from C57BL/6 mice were stained with eFluor 670 **(A)** or unstained **(B,C)**. They were then stimulated with LPS and IL-4 in the absence or presence of SDMCs from sanroque mice, transwells (TW), or inhibitors, as indicated, and assayed by FACS. Ratios of B cells: SDMCs were 1:1 **(A)** and 2:1 **(B,C)**. **(D)** PCs from sanroque mice were cultured with or without SDMCs at a ratio of 2:1 for 48 h and assayed by FACS gated on PCs to detect apoptotic PCs. Floating bars (min to max, line at mean) represent percentages of apoptotic PCs. **(E)** Supernatants were assayed by ELISA to detect IgG. Results from 3 independent experiments are shown. **(F,G)** CD11b^+^Gr-1^hi^ and CD11b^+^Gr-1^lo^ subsets of SDMCs were separately sorted and co-cultured with PCs as in **(D)**. FACS profiles gated on PCs and plots displayed as means ± SDs are shown. **(H,I)** Sanroque mice were assayed by FACS to measure percentages of PCs, SDMCs and long-lived PCs in spleens, followed by correlation analysis. The data are representative of more than 3 independent experiments. *p < 0.05, **p < 0.01, and ***p < 0.001 by paired **(E)** or unpaired [all except **(E)]** Student's *t*-tests.

To determine the effect of SDMCs from sanroque mice on the activity of B cells, we cocultured syngeneic B cells with SDMCs from sanroque mice aged ~20 weeks. The presence of SDMCs slightly, but significantly, stimulated the LPS- or CD40L-mediated proliferation of B cells (Figure 5A, Supplementary Figure 9). Moreover, the SDMCs led to upregulation of the costimulators CD80 and CD86 on the surface of B cells (Figure 5B). SDMCs also promoted the emergence of surface IgG1-expressing B cells, and this effect was independent of contact, iNOS and arginase-1 (Figure 5C). There was no evidence that the presence of SDMCs stimulated the differentiation of B cells into CD138^+^ PCs under these culture conditions.

Because both SDMCs and PCs are located in extrafollicular areas of the spleen, the SDMCs may interact with PCs to affect their physiology. Our finding that sanroque mice had long-lived PCs that accumulated in their spleens during disease progression (Supplementary Figure 11) suggested that SDMCs might be implicated in this phenomenon. To examine this possibility, we sorted B220^lo^CD138^+^ PCs from sanroque mice, co-cultured them with autologous SDMCs in the presence or absence of LPS, and then assayed for apoptotic cells. The proportions of early and late apoptotic PCs (Annexin V^+^ 7-AAD^−^ and Annexin V^+^ 7-AAD^+^, respectively) were significantly reduced when SDMCs were added to the cultures regardless of the presence or absence of LPS (Figure 5D, Supplementary Figure 11). Consistent with this, supernatants of the cultures with SDMCs contained higher amounts of total IgG than those without SDMCs (Figure 5E). PC survival was promoted to similar extents by the CD11b^+^Gr-1^hi^ and CD11b^+^Gr-1^lo^ subsets of SDMCs (Figure 5F). Since myeloid cell leukemia 1 (MCL1), a prosurvival member of the BCL2 family, is essential for the survival of PCs (55), we measured MCL1 in PCs co-cultured with SDMCs and found that both CD11b^+^Gr-1^hi^ and CD11b^+^Gr-1^lo^ subsets of SDMCs upregulated the expression of MCL1 in PCs (Figure 5G). Finally, we found that the proportion of SDMCs was correlated not only with the proportion of PCs but also with the proportion of long-lived PCs within the whole PC population in sanroque mice (Figures 5H,I). These results taken together demonstrate that SDMCs promote the survival of PCs.

### SDMCs Produce BAFF to Promote Survival of PCs

**Figure 6:**
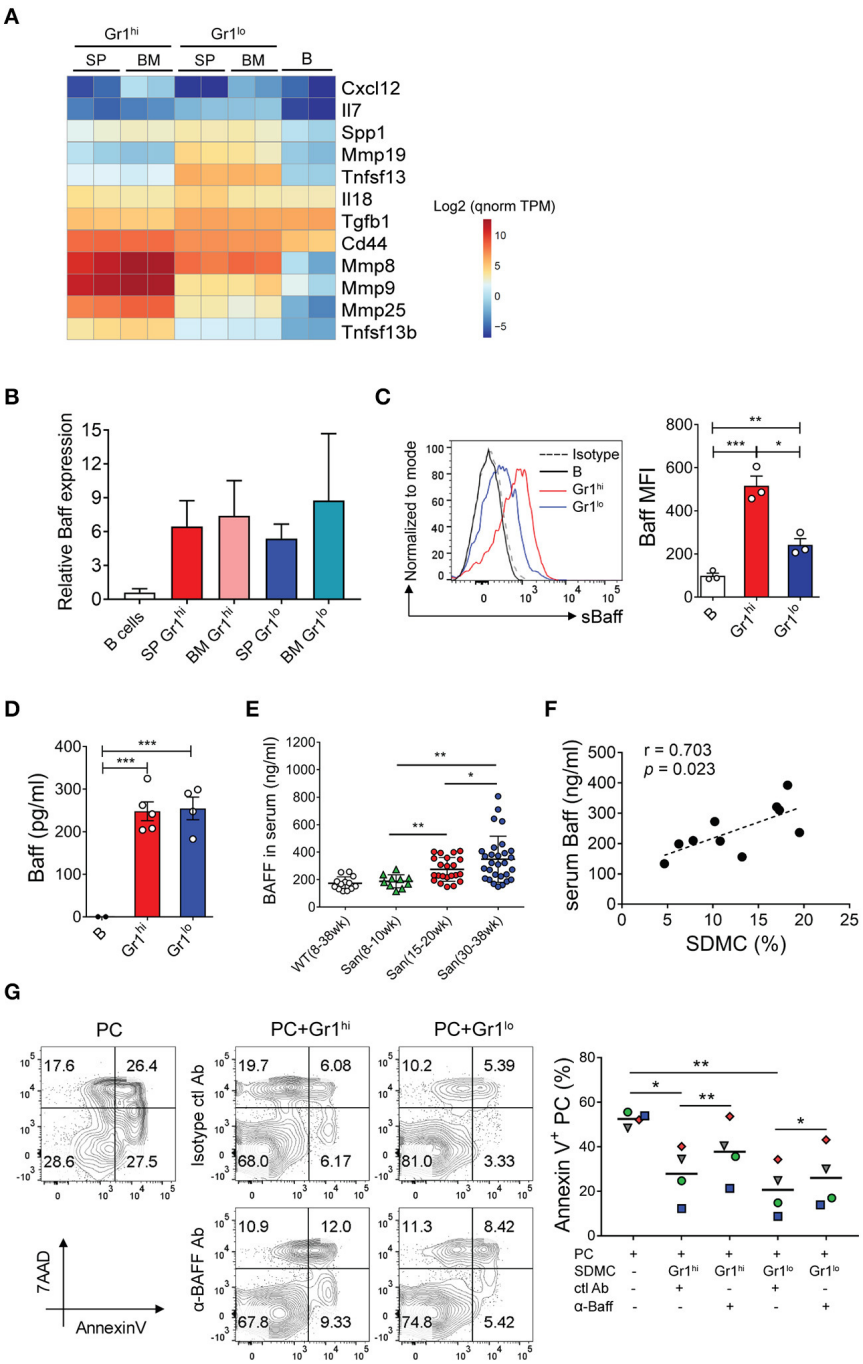
BAFF-mediated effects of SDMCs on PC survival. **(A)** Spleen- and BM-derived CD11b^+^Gr-1^hi^ and CD11b^+^Gr-1^lo^ cells from sanroque mice and WT CD19^+^ B cells were assayed by RNA-seq. Differentially-expressed genes which encode known PC survival factors are displayed as a heatmap. The quantile-normalized (qnorm) TPM values were scaled in the logarithm with base 2 and are presented based on the color-code in the legend. **(B)** These cells were assayed by quantitative RT-PCR. **(C)** The SDMC subsets were analyzed by FACS to detect surface BAFF. Representative FACS profiles and mean fluorescence intensities (MFI) are displayed as means ± SDs with symbols representing values of individual mice. **(D)** The SDMC subsets were cultured in the presence of 1 μg/ml LPS for 48 h and supernatants were assayed by ELISA to detect soluble BAFF. **(E)** Sera were collected from WT and sanroque mice at the indicated ages and analyzed by ELISA. **(F)** Serum levels of BAFF and percentages of SDMCs in sanroque mice were analyzed by correlation analysis. **(G)** CD11b^+^Gr-1^hi^ and Gr-1^lo^ subsets of SDMCs were separately sorted and co-cultured with PCs in the presence of anti-BAFF or isotype-matched control mAb, and assayed by FACS after 48 h. FACS profiles gated on PCs and plots indicating the values of individual samples with means (horizontal lines) are shown. The data are representative of 2 **(B)** or 4 **(C–G)** independent experiments. Each symbol represents an individual experiment. *p < 0.05, **p < 0.01, and ***p < 0.001 by paired **(G)** and unpaired **(C–E)** Student's *t*-test.

The longevity of PCs is largely dependent on the presence of survival niches in their microenvironment. We hypothesized that SDMCs produce survival factors for PCs and thereby establish a niche in autoimmune spleens. *In silico* analysis revealed that SDMC and BM-derived MDSC subsets express many genes that encode PC survival factors at levels higher than do syngeneic WT B cells (Figure 6A). Interestingly, these genes could be roughly divided into two groups, depending on the cell type in which they were activated. The first group of genes was predominantly activated in CD11b^+^Gr-1^lo^ subsets, and encode TGF-β1, IL-18, APRIL (*Tnfsf13*), and more. The second group of genes, which was predominantly activated in CD11b^+^Gr-1^hi^ subsets, includes genes encoding CD44 and BAFF (*Tnfsf13b*).

In view of the evidence that the emergence of splenic long-lived PCs coincides with upregulation of BAFF in patients with human autoimmune disease (14), we asked whether BAFF was the factor linking SDMCs and PCs. Consistent with the RNA-seq data, both CD11b^+^Gr-1^hi^ and CD11b^+^Gr-1^lo^ subsets derived from the spleen and BM of sanroque mice expressed 5 to 8-fold higher levels of BAFF mRNA than autologous B cells (Figure 6B). BAFF protein was detectable on the surfaces of both subsets of SDMCs and also in soluble form in culture supernatants (Figures 6C,D). Serum concentrations increased progressively with age only in sanroque not WT mice, so they became significantly different from 15 to 20 weeks of age (Figure 6E). Accordingly, the serum concentrations of BAFF correlated with the percentages of SDMCs in sanroque mice (Figure 6F). To test whether the effect of SDMCs on PC survival was mediated by BAFF, we added anti-BAFF neutralizing Ab to co-cultures of PCs with each subset of SDMCs. We found that the anti-BAFF Ab partially, but significantly, interfered with the effect of both subsets of SDMCs on PC survival (Figure 6G). Therefore, these results demonstrate that the mechanism by which SDMCs promote the survival of PCs includes the provision of BAFF to PCs in membrane-bound or soluble form.

### Selective Depletion of SDMCs Reduces Humoral Autoimmunity

**Figure 7:**
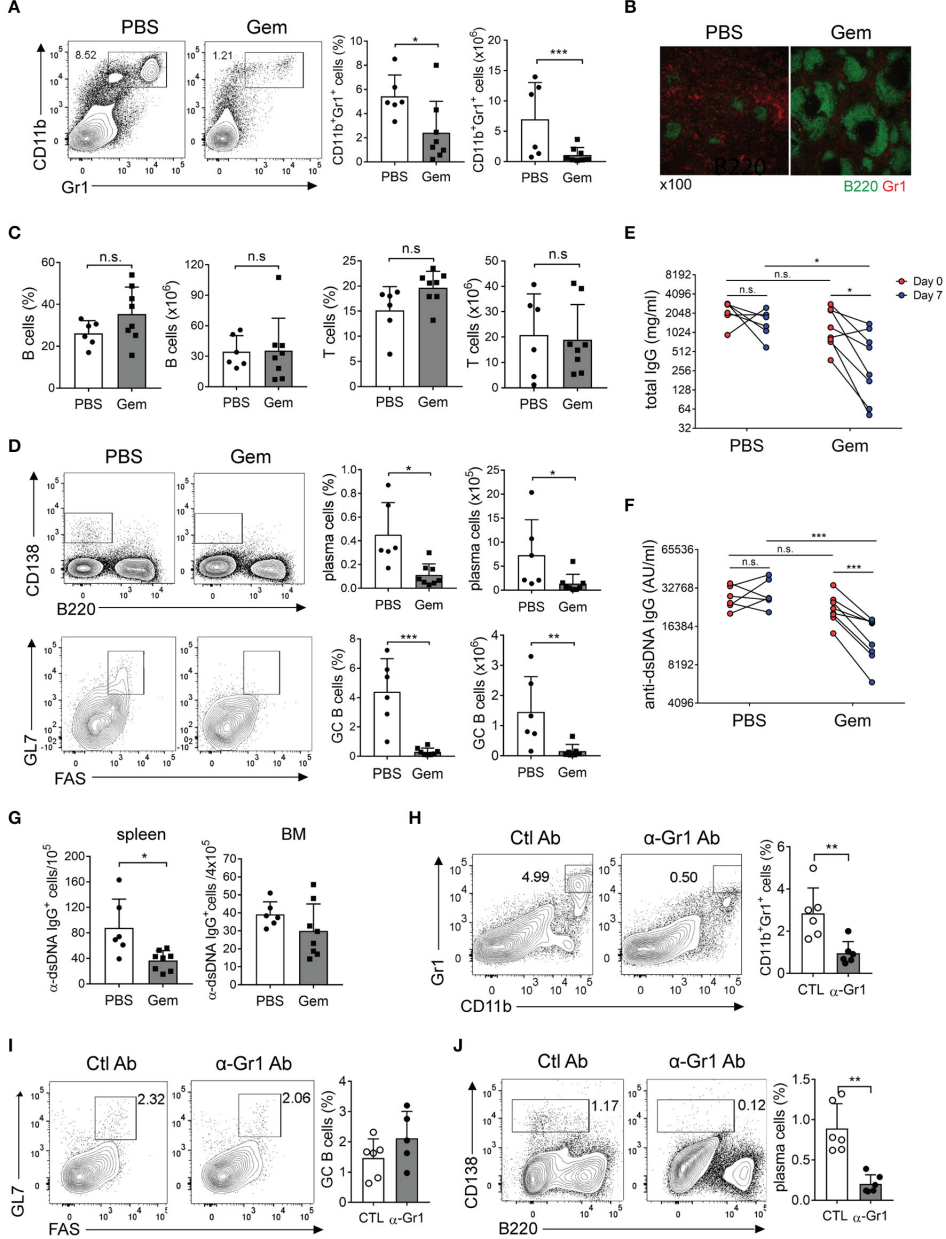
The effect of Gr-1^+^ cell depletion on humoral autoimmunity. **(A–G)** Sanroque mice were injected with gemcitabine (Gem) or PBS and assayed *post-mortem*. **(A)** Representative FACS profiles and plots displaying percentages and numbers of Gr-1^+^ cells are shown. **(B)** Spleen sections were examined by fluorescence confocal microscopy. Original magnification, ×100. **(C)** Percentages and absolute numbers of splenic B and T cells. (D) Representative FACS profiles with percentages of PCs (B220^−^CD138^+^) among total splenocytes, and GC B cells (GL7^+^FAS^+^) among B cells, are shown. **(E,F)** Sera collected on days 0 and 7 were analyzed by ELISA to measure levels of total and anti-dsDNA IgG Abs. **(G)** Splenocytes and BM cells were assayed by ELISPOT to determine numbers of anti-dsDNA-specific IgG Ab-secreting cells. **(H–J)** Disease-induced SKG mice were injected with anti-Gr-1 Ab or isotype-matched control Ab for 4 weeks and assayed by FACS. Representative FACS profiles with percentages of indicated cells are shown. Data are pooled from 2 repeat experiments and displayed as means ± SDs with symbols indicating individual mice. *p < 0.05, **p < 0.01, and ***p < 0.001 by paired **(E,F)** or unpaired (the others) Student's *t*-tests. San, sanroque.

To confirm the effect of SDMCs on autoantibody responses *in vivo* we took advantage of gemcitabine, which is known to specifically delete Gr-1^+^ cells (32, 56). We treated disease-established sanroque mice with gemcitabine or vehicle for 1 week and assessed Ab responses. We confirmed first that gemcitabine treatment significantly reduced the frequency of CD11b^+^Gr-1^+^ cells in the spleen (Figure 7A). Gr-1^+^ cells almost disappeared in the spleens of gemcitabine-treated mice observed by fluorescence microscope (Figure 7B). This reduction seemed to be specific, because the relative sizes of T and B cell populations were not altered (Figure 7C). Importantly, the proportions and numbers of B220^lo^CD138^+^ PCs and B220^+^FAS^+^GL7^+^ GC B cells were dramatically decreased in the spleens of gemcitabine-treated mice (Figure 7D). Consistent with this, titers of total IgG and anti-dsDNA IgG Ab were also significantly decreased by gemcitabine treatment (Figures 7E,F). Cells secreting anti-dsDNA IgG were significantly fewer in the spleens of gemcitabine-treated mice than in those of PBS-treated mice (Figure 7G). The BM contained ~8 times fewer cells secreting this Ab, with no difference between the two groups. These results suggested that depletion of SDMCs resulted in dampened Ab responses, but the potential cytotoxic effect of gemcitabine on rapidly proliferating GC B cells could not be ruled out in this system.

To rule out this possibility, we conducted additional experiments using Ab-mediated depletion methods. SKG mice at the early autoimmune phase were repetitively injected with anti-Gr-1 Ab for 4 weeks, which was sufficient to significantly reduce the fraction of Gr-1^+^ cells without altering the frequency of GC B cells (Figures 7H,I). Importantly, treatment of anti-Gr-1 Ab significantly reduced the proportion of B220^−/lo^CD138^hi^ PCs (Figure 7J). Thus, these results further support the idea that SDMCs play a role in exacerbating Ab-mediated autoimmunity.

## Discussion

**Figure 8:**
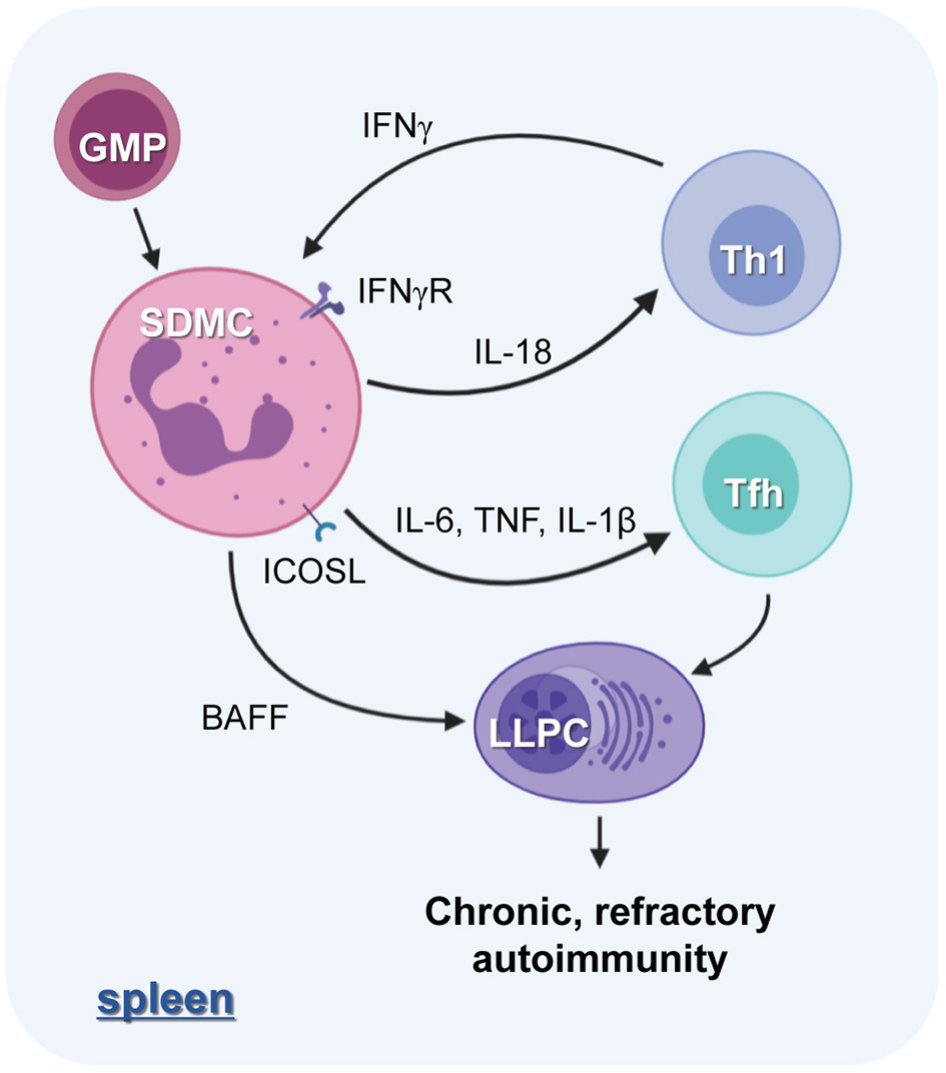
The role of SDMCs in lupus autoimmunity. The proinflammatory cytokine milieu of the spleens of disease-established sanroque mice promotes the *in situ* expansion and pathologic activation of SDMCs. The SDMCs promote the development of Th1 and Tfh cells. Th1 cells, in turn, exert positive feedback on the emergence of SDMCs, and the Tfh cells are required for the development of long-lived PCs (LLPCs). SDMCs directly support the maintenance of LLPCs by providing BAFF, and the LLPCs cause the disease to be chronic and refractory to cytostatic treatments.

We have shown above that SDMCs expand *in situ* during the progression of autoimmune disease and exacerbate autoimmunity. Phenotypically, SDMCs are a mixture of G-MDSCs and M-MDSCs in which proinflammatory signals are more strongly activated than in their BM counterparts. They seem to develop in the spleen through extramedullary myelopoiesis and then acquire pathological phenotypes. These processes may occur because the milieu is rich in cytokines, such as GM-CSF, IL-6, and IFN-γ, produced in part by CD4^+^ T cells. Functionally, although the spleen-derived MDSCs fit the definition of that term, namely they are “suppressive” to the proliferation of T cells, we found that they are much more than their name suggests. Surprisingly, they promote the differentiation of CD4^+^ T cells to IFN-γ-secreting Th1 cells and humoral effector Tfh cells. Given that IFN-γ signals are crucial for the pathologic activation of MDSCs, the SDMCs seem to license CD4^+^ T cells to be potent stimulators of their pathologic conversion. In addition, since IFN-γ signals can activate autoreactive B cells (57, 58), the SDMC-Th1 axis may indirectly contribute to autoantibody responses. Apart from elevating Ab responses through Tfh cells, SDMCs directly interact with B cells and PCs to increase their pathologic status. The B cells that have interacted with SDMC upregulate B7 costimulators and give rise to more numerous IgG^+^ post-switched cells, while the PCs acquire longer life spans in a process that is partially dependent on the provision of BAFF by the SDMCs. Thus, *in situ* crosstalk between the myeloid-derived cells and diverse lymphoid-derived humoral effector cells underpins the progressive exacerbation of autoimmunity, as depicted in Figure 8. Our evidence that breaking this inflammatory circuit by depleting SDMCs attenuates autoimmunity further supports this idea.

In contradiction to our results, most previous studies have demonstrated suppressive effects of MDSCs on the differentiation of Th1 and Tfh cells. Specifically, splenic MDSCs suppressed the differentiation of Th1 cells in murine models of pristane-induced lupus, collagen-induced arthritis and experimental autoimmune encephalomyelitis (EAE); they also inhibited the differentiation of Tfh cells in (NZBxNZW)F1 mice and mice infected with Japanese encephalitis virus (20, 30, 32–34). The effects of MDSCs on B cells are even more controversial since MDSCs have been found to be suppressive in young male (NZBxNZW)F1 mice, collagen-induced arthritic mice, and EAE-established mice, and supportive in old male and female (NZBxNZW)F1 mice and tumor-bearing mice (20, 31, 35, 36, 59). Our results obtained in the context of *in vitro* B cell culture are in line with the latter findings.

There are several possible explanations for this discrepancy. First, it may be due to the heterogeneous nature of MDSCs. Despite its subdivision into two major subsets, the MDSC population is composed of a continuum of immature myeloid cells with diverse phenotypes and activities. The cellular composition of MDSCs could differ depending on the experimental system, and this might lead to diverse outcomes. Second, due to their phenotypic similarity, it is difficult to discriminate MDSCs from neutrophils and monocytes, and so the identities of cells used in different studies may be different. For example, splenic CD11b^+^Ly6G^hi^ cells that emerged in the same murine lupus model were identified as neutrophils or G-MDSCs in two different studies (27, 59). Because G-MDSCs and neutrophils are functionally distinct (26, 27, 60), we reasoned that discrimination between them was important, and we performed transcriptome analyses that revealed that the SDMCs were authentic MDSCs, not neutrophils or monocytes. Finally, the functional diversity may reflect the plastic nature of MDSCs (61). A good example comes from (NZBxNZW)F1 mice, in which CD11b^+^Gr-1^lo^ cells are suppressive to the development of Tfh cells at early times, but later promote the development of PCs (20, 59). Our data showed that the SDMCs have a transcriptome profile different from BM-derived MDSCs, thus illustrating a further kind of MDSC plasticity. Specifically, the SDMCs displayed a more proinflammatory phenotype with enhanced responses to cytokines such as TNF, IFN-α, IFN-γ, IL-6, and IL-2. In this regard, we speculate that this phenotype is evoked by the proinflammatory cytokine milieu of the spleen.

In support of this idea, we found that lupus-established sanroque mice overexpress GM-CSF, IL-6, and IFN-γ in their spleens, and CD4^+^ T cells are in part responsible for their production. Given that these cytokines were found to be crucial for triggering the expansion (GM-CSF and IL-6) or pathologic activation (IFN-γ and IL-6) of MDSCs in studies with tumor tissue or BM stroma (24, 25), the splenic microenvironment of sanroque mice may be sufficient to serve as a niche for SDMCs. Inside the niche, splenic extramedullary myelopoiesis may be abnormally activated and thus biased toward the MDSC fate rather than the mature cell fate as in classical myelopoiesis. In this regard, we postulate that the SDMCs originate from the spleen rather than migrating from the BM. The positive feedback loop that we identified between SDMCs and CD4^+^ T cells may act as the driving force for this process.

Although SDMCs are distinct from typical neutrophils, they are reminiscent of aberrant subsets of neutrophils in some aspects, which are enriched in the spleens of patients with SLE. One example is a subset of low-density granulocytes (LDGs) (62), in which phagocytic capacity is impaired and the cytotoxicity to endothelial cells is mediated by type I IFNs. LDGs have a proinflammatory phenotype along with the capacity to synthesize IFN-α, and these features were also shown in the Gr-1^lo^ subset of SDMCs. Nevertheless, the Gr-1^lo^ SDMC does not exactly phenocopy the LDG, since the former has a CD86^+^MHCII^+^CD115^+/low^ phenotype, while the later has a CD86^−^MHCII^−^CD115^−^ phenotype. Moreover, cellular compositions based on nuclear morphology are different; the majority of the former and the latter has a monocyte- and PMN-like appearance, respectively. We also need to check out whether Gr-1^hi^ SDMCs are a murine model of human splenic neutrophils, so-called marginal zone B cell-helper neutrophils (N_BH_) (63). These cells were identified at peri-marginal zone areas of spleen in humans under homeostatic conditions, but widely infiltrated into diverse areas of spleen at pathologic states including SLE. We found some functional similarities between Gr-1^hi^ SDMCs and N_BH_, such as functioning as B cell helpers by providing BAFF and suppressing proliferation of CD4^+^ T cells. However, there are many differences between two populations. For example, N_BH_ are derived from circulating neutrophils—after homing to spleen, they are reprogrammed to N_BH_ in a manner dependent on sinusoidal endothelial cells. However, Gr-1^hi^ SDMCs appear to emerge *in situ* from splenic myeloid progenitors. The suppressive mechanism is contact-independent and -dependent in N_BH_ and Gr-1^hi^ SDMCs, respectively. Most importantly, we have revealed that Gr-1^hi^ SDMCs prolong the lifespan of PCs, which was not elucidated as a role of N_BH_.

How can SDMCs promote the differentiation of CD4^+^ T cells into Th1 and Tfh cells? The underlying mechanism remains largely unknown, but our transcriptome and gene expression analyses may provide one clue to the answer. Because SDMCs highly expressed molecules known as inducers of Th1 cells (IL-18) and Tfh cells (IL-6, IL-1β, TNF, and ICOSL) (64–67), we reasoned that part of the mechanism should be made up by those molecules. Indeed, the data demonstrating the effect of anti-IL-18 Ab on SDMC/T co-culture supported this idea. Whether the other molecules are indeed involved in the mechanism is currently under investigation.

We and others have provided evidence that long-lived PCs accumulate in the spleens of autoimmune mice, and are deleterious because of their persistent Ab production and resistance to cytostatic treatment (11). Furthermore, we have revealed that cell-autonomous mechanisms lead them to survive under immune complex-rich milieu and proteotoxic states and to establish a positive feedback loop with Tfh cells (11, 15, 16). Nevertheless, little is known about the types of cells that are required to support the survival of splenic PCs, as eosinophils are needed for BM long-lived PCs (68). Here we found that SDMCs are the key providers of PC survival factors including BAFF. Therefore, we propose that SDMCs are the cells contributing to the establishment of a niche for the long-term survival of PCs in autoimmune spleens. Interestingly, this result is consistent with previous reports that the accumulation of splenic long-lived PCs was associated with local production of BAFF in the spleens of patients with autoimmune disease (14, 69). We reasoned that IFN-α-triggerred signals would be involved in the induction of BAFF by SDMCs, because IFN-α can induce BAFF expression in myeloid cells *in vitro* (70), and genes related to IFN-α signals are highly activated in both the G-MDSC and M-MDSC subsets of SDMCs, according to our transcriptome analysis. We thus have provided what we believe to be the first evidence of a physical and functional link between SDMCs (i.e., spleen-derived MDSCs) and PCs. Our result also suggests that interventions targeting the SDMCs could provide a new therapeutic strategy for removing long-lived splenic PCs that are resistant to cytotoxic drugs.

In conclusion, we have shown, first, that during lupus progression SDMCs emerge via splenic extramedullary myelopoiesis and undergo pathologic activation *in situ*. Second, SDMCs and CD4^+^ T cells establish an inflammatory positive feedback loop. Finally, SDMCs amplify autoantibody responses by supporting Tfh cell induction, B cell activation, and PC survival. Thus, our study provides insight into the mechanisms by which humoral immunity is hyperactivated in systemic autoimmune diseases.

## Data Availability Statement

The datasets presented in this study can be found in online repositories. The names of the repository/repositories and accession number(s) can be found here: https://www.ncbi.nlm.nih.gov/geo/query/acc.cgi?acc=GSE161850.

## Ethics Statement

The animal study was reviewed and approved by the Institutional Animal Care and Use Committee of Hanyang University.

## Author Contributions

EJ and SC performed experiments and prepared figures. EJ and JY designed experiments and interpreted data. SP and J-WN analyzed RNA-seq data and interpreted the results. EJ, J-WN, and JY wrote the manuscript. JY supervised the study. All authors reviewed the manuscript.

## Conflict of Interest

The authors declare that the research was conducted in the absence of any commercial or financial relationships that could be construed as a potential conflict of interest.

## Abbreviations

7-AAD: 7-aminoactinomycin D
BAFF: B-cell activating factor of the TNF family
C/EBP: CCAAT/enhancer-binding protein
DEG: differentially expressed gene
dsDNA: double-stranded DNA
FC: fold change
GC: germinal center
G-MDSC: granulocytic myeloid-derived suppressor cell
GMP: granulocyte-macrophage progenitor
iNOS: inducible nitric oxide synthase
LT-HSC: long term-hematopoietic stem cell
mAb: monoclonal antibody
MCL1: myeloid cell leukemia 1
MDSC: myeloid-derived suppressor cell
MEP: megakaryocyte-erythrocyte progenitor
M-MDSC: monocytic myeloid-derived suppressor cell
MPP: multipotent progenitor
PC: plasma cell
RNA-seq: RNA sequencing
SDMC: spleen-derived CD11b^+^Gr-1^+^ myeloid cell
SLE: systemic lupus erythematosus
Tfh: follicular helper T
WT: wild-type.
