# Supplementary material for: An Inflammatory Loop Between Spleen-Derived Myeloid Cells and CD4+ T Cells Leads to Accumulation of Long-Lived Plasma Cells That Exacerbates Lupus Autoimmunity
Source: Front Immunol. 2021 Feb 11;12:631472. doi: 10.3389/fimmu.2021.631472 (PMC7904883; doi:10.3389/fimmu.2021.631472)
Supplement: Supplementary file 1 [file Data_Sheet_1.PDF]

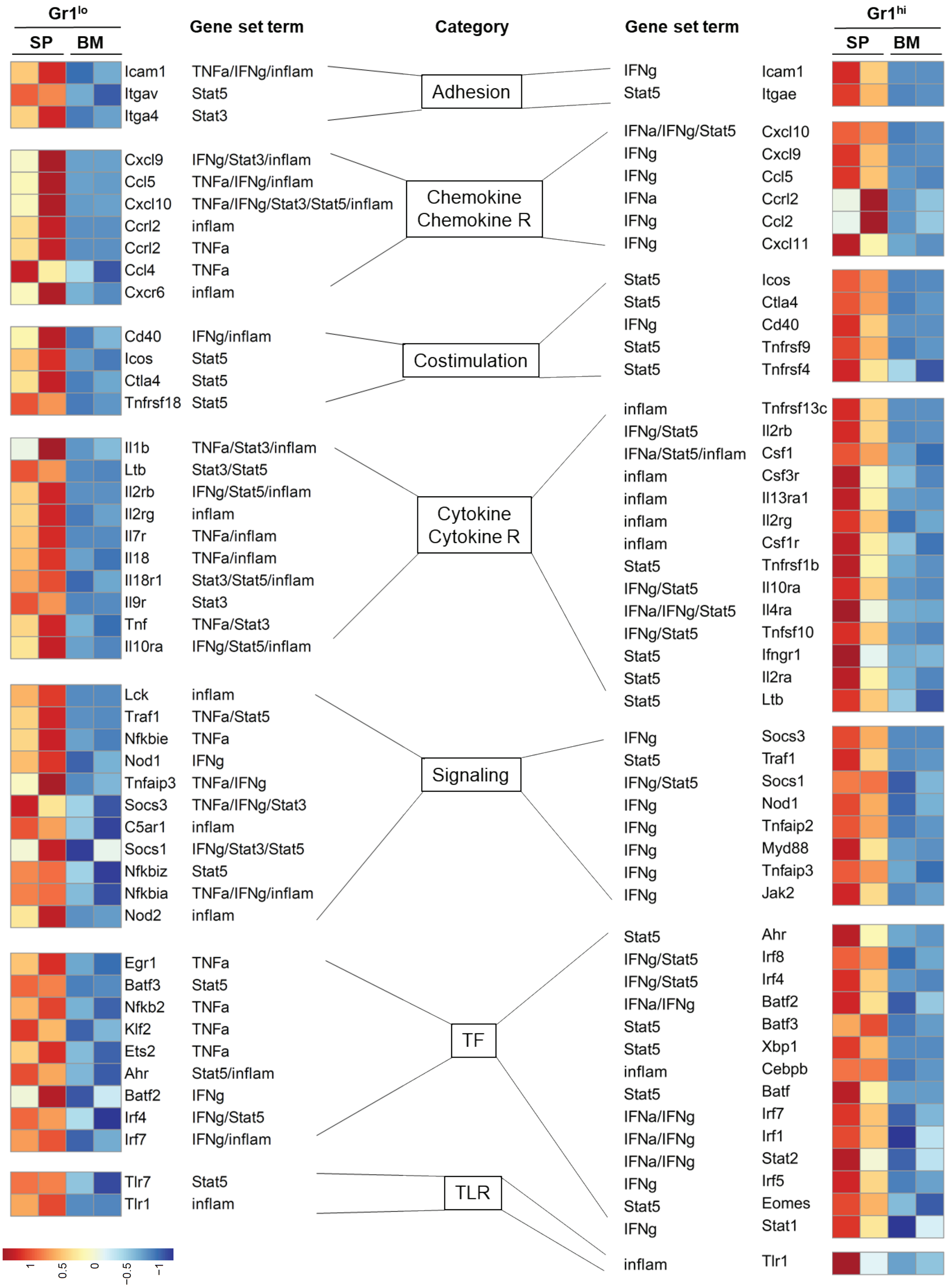

Fig. S1. RNA-seq data of SDMCs. Heatmaps of upregulated genes in Gr-1^{lo} and Gr-1^{hi} SDMCs compared to BM-derived counterparts. The genes are included in the significantly enriched gene set terms in GSEA. The enriched gene set terms include IFN-α response (IFNa), IFN-γ response (IFNg), IL-2-STAT5 signaling (STAT5), TNF-α signaling via NF-κB (TNFa), inflammatory response (inflam), IL-6-JNK-STAT3 signaling (STAT3). The genes are also categorized according to modes of their activities. R, receptor; TF, transcription factor.
