# Supplementary material for: An Inflammatory Loop Between Spleen-Derived Myeloid Cells and CD4+ T Cells Leads to Accumulation of Long-Lived Plasma Cells That Exacerbates Lupus Autoimmunity
Source: Front Immunol. 2021 Feb 11;12:631472. doi: 10.3389/fimmu.2021.631472 (PMC7904883; doi:10.3389/fimmu.2021.631472)
Supplement: Supplementary file 2 [file Data_Sheet_2.PDF]

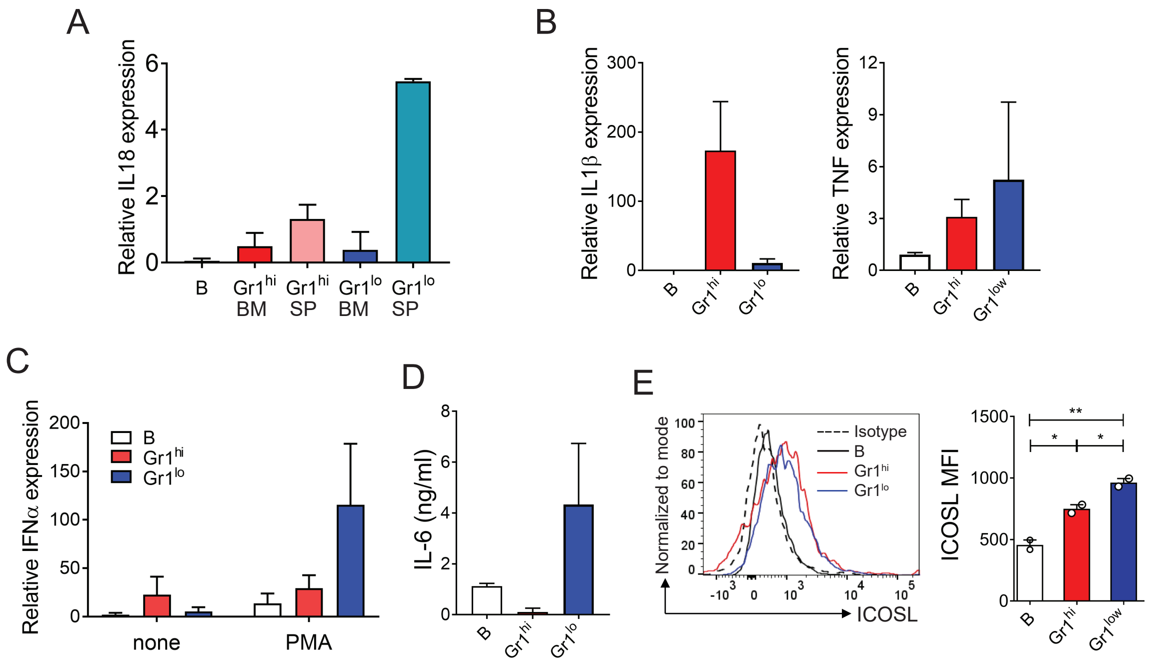

Fig. S2. Upregulation of proinflammatory molecules in SDMCs. Gr-1hi and Gr-1lo SDMC subsets and autologous B and BM counterpart cells were sorted and assayed by qRT-PCR (A and B) and FACS (E). The sorted cells were cultured with or without 20 ng/ml PMA for 3 hours and assayed by qRT-PCR (C). The sorted cells were cultured in the presence of 2 µg/ml LPS for 48 hours and assayed by ELISA (D).
