# Supplementary material for: An Inflammatory Loop Between Spleen-Derived Myeloid Cells and CD4+ T Cells Leads to Accumulation of Long-Lived Plasma Cells That Exacerbates Lupus Autoimmunity
Source: Front Immunol. 2021 Feb 11;12:631472. doi: 10.3389/fimmu.2021.631472 (PMC7904883; doi:10.3389/fimmu.2021.631472)
Supplement: Supplementary file 3 [file Data_Sheet_3.PDF]

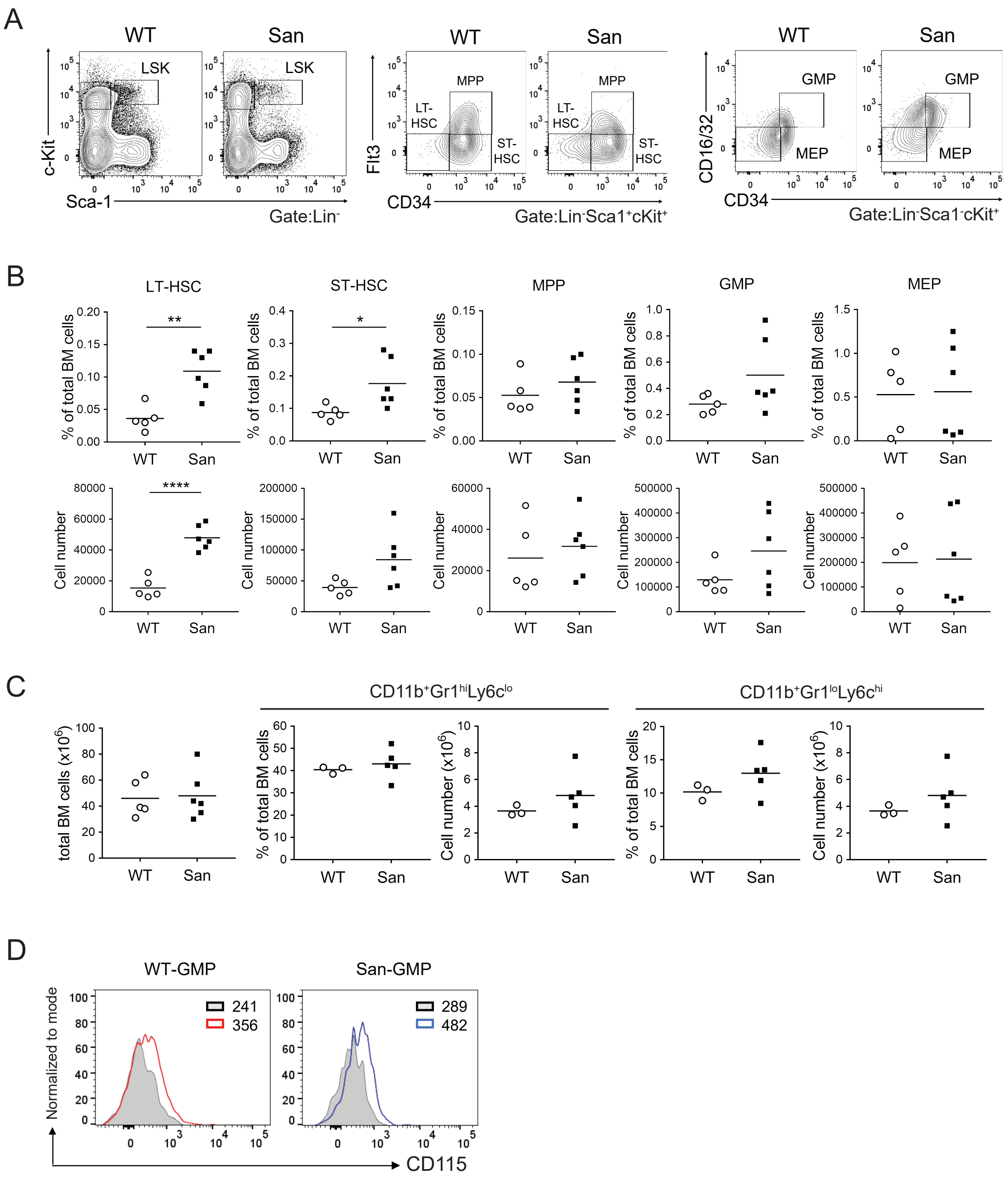

Fig. S3. BM cellularity of sanroque mice and CD115 expression in splenic GMPs. (A-C) BM cells from sanroque mice and their WT littermates were assayed by FACS. Representative FACS profiles (A) and percentages and numbers of the indicated cell populations are shown with symbols representing individual mice and horizontal bars representing mean values (B and C). There was no difference in BM cellularity between sanroque mice and their WT littermates. (D) Splenic GMPs from sanroque mice and their WT littermates were assayed by FACS to detect CD115. Representative histograms with mean fluorescence intensities are shown. Gray filled histograms display isotype control staining. The level of CD115 was not significantly different between two groups. *p<0.05, **p<0.01, and ****p<0.0001 by Student's t-tests. San, sanroque.
