# Supplementary material for: An Inflammatory Loop Between Spleen-Derived Myeloid Cells and CD4+ T Cells Leads to Accumulation of Long-Lived Plasma Cells That Exacerbates Lupus Autoimmunity
Source: Front Immunol. 2021 Feb 11;12:631472. doi: 10.3389/fimmu.2021.631472 (PMC7904883; doi:10.3389/fimmu.2021.631472)
Supplement: Supplementary file 4 [file Data_Sheet_4.PDF]

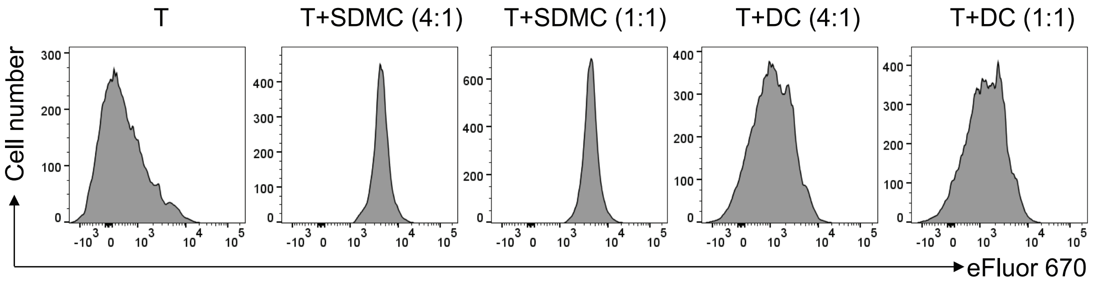

Fig. S4. Functional difference between SDMC and splenic DC. $CD4^+$ T cells were labeled with eFlour 670, stimulated with anti-CD3 and anti-CD28 Abs in the presence or absence of SDMCs or DCs from sanroque mice at the indicated ratios for 3 days, and assayed by FACS.
