# Supplementary material for: An Inflammatory Loop Between Spleen-Derived Myeloid Cells and CD4+ T Cells Leads to Accumulation of Long-Lived Plasma Cells That Exacerbates Lupus Autoimmunity
Source: Front Immunol. 2021 Feb 11;12:631472. doi: 10.3389/fimmu.2021.631472 (PMC7904883; doi:10.3389/fimmu.2021.631472)
Supplement: Supplementary file 5 [file Data_Sheet_5.PDF]

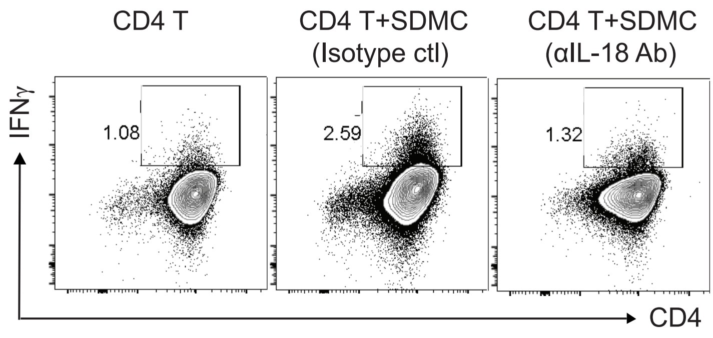

Fig. S5. IL-18 produced by SDMC induces IFNγ production by CD4 T cells. $CD4^{+}$ T cells were stimulated with anti-CD3 and anti-CD28 mAbs in the presence or absence of SDMCs from sanroque mice. Neutralizing Ab to IL-18 or isotype-matched control Ab was added to the culture. The resultant cells were assyed by intracellular FACS. Representative FACS profiles are ahown.
