# Supplementary material for: An Inflammatory Loop Between Spleen-Derived Myeloid Cells and CD4+ T Cells Leads to Accumulation of Long-Lived Plasma Cells That Exacerbates Lupus Autoimmunity
Source: Front Immunol. 2021 Feb 11;12:631472. doi: 10.3389/fimmu.2021.631472 (PMC7904883; doi:10.3389/fimmu.2021.631472)
Supplement: Supplementary file 6 [file Data_Sheet_6.PDF]

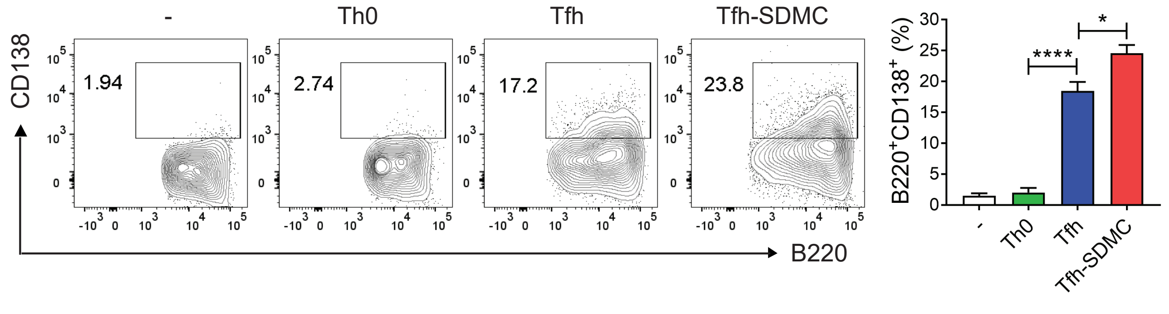

Fig. S6. SDMCs license CD4+ T cells to be effective helpers for B cell differentiation into PCs. CD4+ T cells were cultured under Th0 or Tfh-polarizing conditions in the presence or absence of SDMCs, cocultured with syngeneic B cells in the presence of anti-IgM and IL-4 and assayed by FACS. Data are representative of 3 independent experiments. The B cells gave rise to CD138+ PCs most efficiently when stimulated with CD4+ T cells cultured under Tfh-polarizing conditions in the presence of SDMCs. *$p<0.05$ and ****$p<0.0001$ by Student's *t*-tests.
