# Supplementary material for: An Inflammatory Loop Between Spleen-Derived Myeloid Cells and CD4+ T Cells Leads to Accumulation of Long-Lived Plasma Cells That Exacerbates Lupus Autoimmunity
Source: Front Immunol. 2021 Feb 11;12:631472. doi: 10.3389/fimmu.2021.631472 (PMC7904883; doi:10.3389/fimmu.2021.631472)
Supplement: Supplementary file 7 [file Data_Sheet_7.PDF]

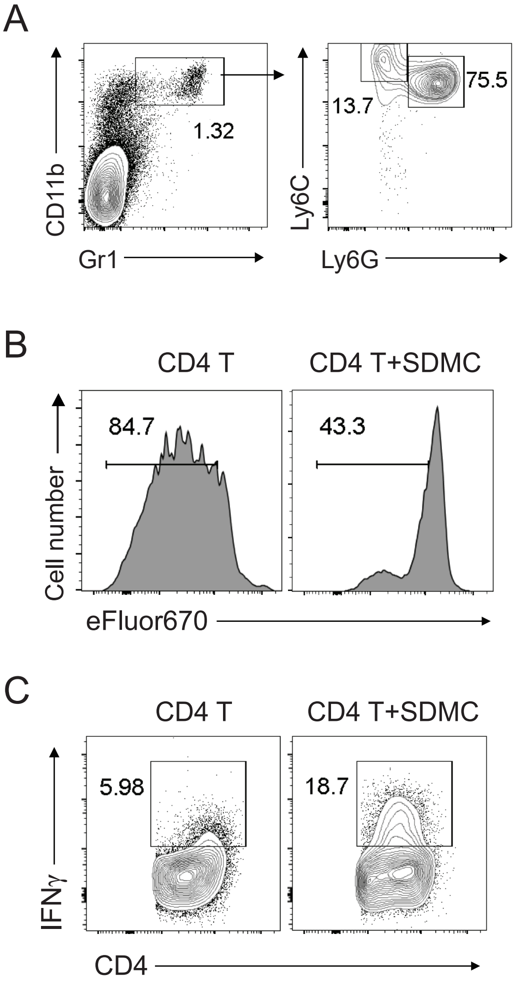

Fig. S7. SDMCs from MRL^{lpr} mice promote the differentiation of CD4^{+} T cells to Th1 cells, while retaining their suppressive activity. (A) Spleen cell from approximately 20-wk-old female MRL^{lpr} mice were assayed by FACS. FACS profiles gated on whole live cells and percentages of cells within the indicated areas are shown. (B) CD4^{+} T cells from MRL^{lpr} mice were stained with eFluor 670 and stimulated with anti-CD3 and anti-CD28 mAbs in the presence or absence of autologous SDMCs at an equal ratio. (C) CD4^{+} T cells were cultured under Th1-polarizing conditions in the presence or absence of SDMCs and assayed by intracellular FACS.
