# Supplementary material for: An Inflammatory Loop Between Spleen-Derived Myeloid Cells and CD4+ T Cells Leads to Accumulation of Long-Lived Plasma Cells That Exacerbates Lupus Autoimmunity
Source: Front Immunol. 2021 Feb 11;12:631472. doi: 10.3389/fimmu.2021.631472 (PMC7904883; doi:10.3389/fimmu.2021.631472)
Supplement: Supplementary file 8 [file Data_Sheet_8.PDF]

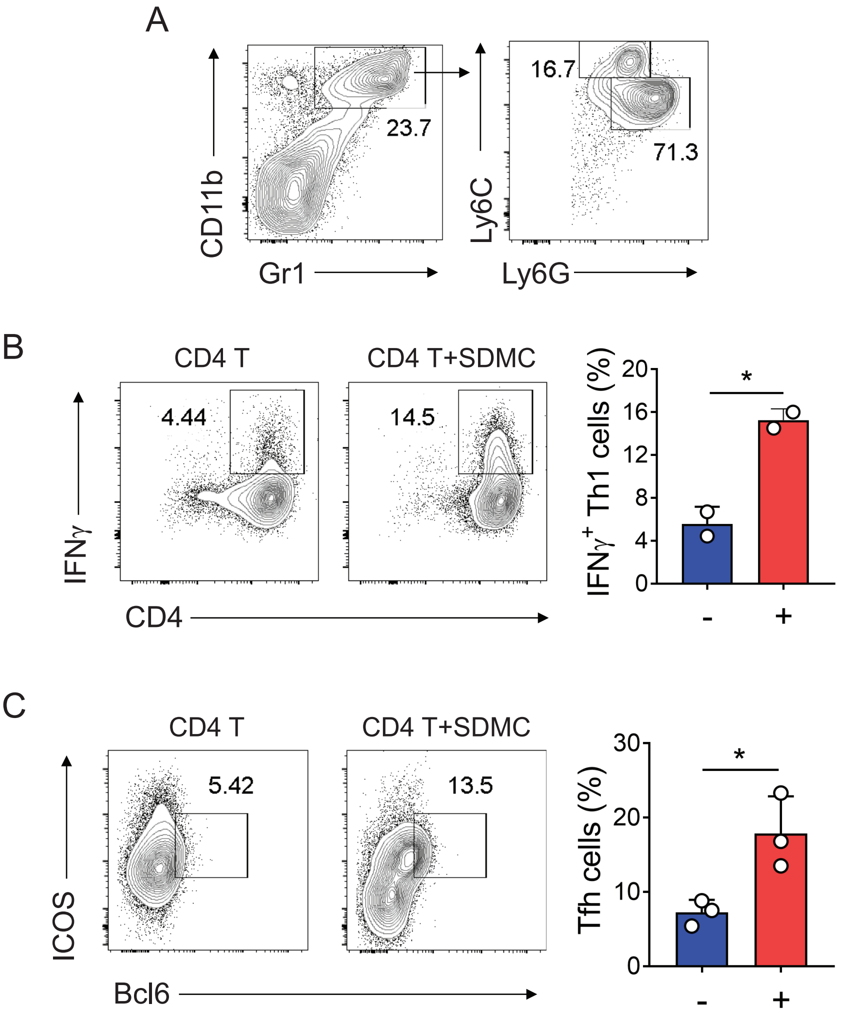

Fig. S8. SDMCs are accumulated in autoimmunity-established SKG mice and promote the differentiation of CD4$^+$ T cells to Th1 and Tfh cells. SKG mice were injected intraperitoneally with curdlan (Wako, Osaka, Japan) at a dose of 3 mg/mouse to induce arthritis and assayed post mortem 5 wks post-injection. (A) Spleen cells were assayed by FACS. FACS profiles gated on whole live cells and percentages of cells within the indicated areas are shown. (B-C) CD4$^+$ T cells were cultured under Th1- or Tfh-polarizing conditions in the presence or absence of SDMCs and assayed by intracellular FACS.
