# Supplementary material for: An Inflammatory Loop Between Spleen-Derived Myeloid Cells and CD4+ T Cells Leads to Accumulation of Long-Lived Plasma Cells That Exacerbates Lupus Autoimmunity
Source: Front Immunol. 2021 Feb 11;12:631472. doi: 10.3389/fimmu.2021.631472 (PMC7904883; doi:10.3389/fimmu.2021.631472)
Supplement: Supplementary file 9 [file Data_Sheet_9.PDF]

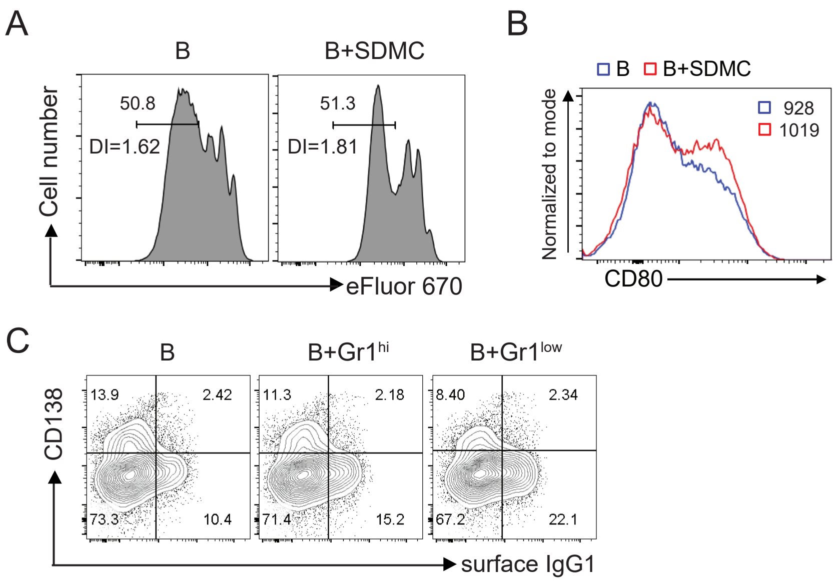

Fig. S9. Effects of SDMCs on CD40L-mediated activation of B cells. eFluor 670-labeled B cells from C57BL/6 mice were cultured with CD40L and IL-4 in the presence or absence of whole SDMCs (A and B) or each subset of SDMCs (C) for 3 (A and B) or 4 (C) days and assayed by FACS. Representative FACS profiles with division index (A), mean fluorescence indensity (B), and percentage of cells in the indicated areas (C) are shown. DI, division index
