# Supplementary material for: An Inflammatory Loop Between Spleen-Derived Myeloid Cells and CD4+ T Cells Leads to Accumulation of Long-Lived Plasma Cells That Exacerbates Lupus Autoimmunity
Source: Front Immunol. 2021 Feb 11;12:631472. doi: 10.3389/fimmu.2021.631472 (PMC7904883; doi:10.3389/fimmu.2021.631472)
Supplement: Supplementary file 10 [file Data_Sheet_10.PDF]

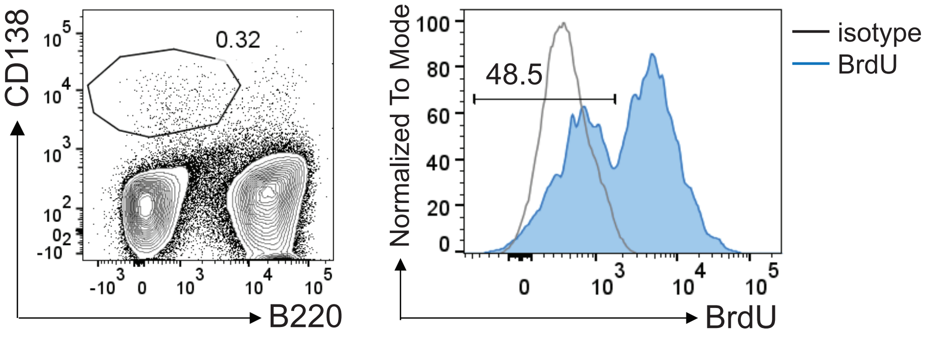


**Fig. S10. Accumulation of long-lived plasma cells in the spleen of sanroque mice.** Sanroque mice aged approximately 20 wk were fed 0.8 mg/ml BrdU (Sigma-Aldrich) in drinking water for 2 wks. After staining with anti-B220 and anti-CD138 Abs, spleen cells were fixed, permeabilized, treated with 50 Kunitz units DNase (Sigma-Aldrich), and stained with FITC-conjugated anti-BrdU mAb (BD Biosciences) according to the manufacturer's instructions, followed by FACS. Representative histograms gated on B220$^{lo}$CD138$^{+}$ PCs are shown. Approximately half of the PCs were BrdU$^{-}$ non-dividing long-lived PCs.
