# Supplementary material for: An Inflammatory Loop Between Spleen-Derived Myeloid Cells and CD4+ T Cells Leads to Accumulation of Long-Lived Plasma Cells That Exacerbates Lupus Autoimmunity
Source: Front Immunol. 2021 Feb 11;12:631472. doi: 10.3389/fimmu.2021.631472 (PMC7904883; doi:10.3389/fimmu.2021.631472)
Supplement: Supplementary file 11 [file Data_Sheet_11.PDF]

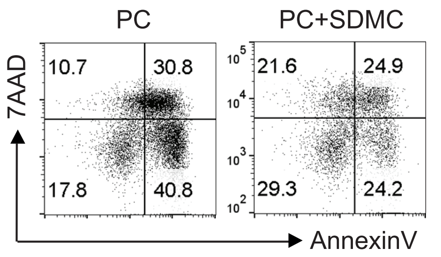

Fig. S11. Effects of unstimulated SDMCs on survival of PCs. PCs from sanroque mice were cultured in the presence or absence of SDMCs at a ratio of 2:1 for 48 hours. Any stimulant (e.g. LPS) was not added to the culture. The cells were assayed by FACS gated on PCs to detect apoptotic PCs.
